# Therapeutic effect of traditional Chinese medicine on diabetic sarcopenia: a systematic review and meta-analysis of preclinical studies

**DOI:** 10.3389/fendo.2025.1647271

**Published:** 2026-01-12

**Authors:** Ruiting Zhang, Yingzhe Liu, Lin Zhu, Dongxia Li, Xiangbin Pan

**Affiliations:** 1 Heilongjiang University of Chinese Medicine, Harbin, China; 2 The Second Affiliated Hospital of Heilongjiang University of Chinese Medicine, Harbin, China

**Keywords:** traditional Chinese medicine, diabetic sarcopenia, preclinical studies, therapeutic effect, meta-analysis

## Abstract

**Background:**

Diabetic sarcopenia (DS) is an emerging complication of diabetes. Although clinical treatment options remain inadequate, preliminary evidence suggests that Traditional Chinese Medicine (TCM) holds therapeutic promise. This study aims to systematically evaluate the existing preclinical evidence for TCM treatments in diabetic sarcopenia.

**Methods:**

We conducted a meta-analysis of animal studies investigating TCM interventions for DS. The initial literature search cutoff date was June 2024, with an updated search conducted during manuscript revision in November 2025. The search covered all records from seven databases from their inception to November 2025. Data analysis is performed using Review Manager 5.3 software. A fixed-effect model was selected when heterogeneity among studies was insignificant (P ≥ 0.1 or I² ≤ 50%); otherwise, a random-effects model was used. The robustness of the primary outcomes was assessed through sensitivity analysis. Publication bias was evaluated using Egger’s test and funnel plots. Additionally, meta-regression was employed to explore potential sources of heterogeneity.This study has been registered on the PROSPERO platform (Registration Number: CRD42024596404).

**Results:**

The meta-analysis demonstrated that TCM intervention significantly increased gastrocnemius muscle weight [SMD = 1.85, 95% CI (1.07, 2.63), P < 0.00001] and muscle cross-sectional area [SMD = 2.68, 95% CI (1.63, 3.73), P < 0.00001], while improving muscle tissue morphology and grip strength [SMD = 1.57, 95% CI (0.49, 2.65), P = 0.004]. Notably, TCM exerted a bidirectional regulatory effect on body weight: significantly increasing it in diabetic cachexia models [SMD = 0.73, P = 0.02] while reducing it in obesity with sarcopenia models [SMD = -1.97, P < 0.00001]. A significant reduction in blood glucose levels was also observed [SMD = -3.13, P < 0.00001]. Mechanistically, preclinical evidence suggests that specific TCM interventions may modulate protein turnover homeostasis, potentially by activating the IGF1-PI3K-Akt-mTOR synthetic pathway and inhibiting the FoxO/Ubiquitin-Proteasome degradation pathway, alongside optimizing the pathological microenvironment.

**Conclusion:**

Current preclinical evidence suggests that specific TCM interventions may show potential in alleviating Diabetic Sarcopenia in animal models by improving muscle mass, strength, and metabolic function. Certain formulations appeared to exhibit a bidirectional regulation of body weight, which may reflect a context-dependent metabolic impact. However, due to the prevalent lack of blinding and allocation concealment in the included studies, the reported efficacy may be systematically overestimated. Consequently, current evidence remains preliminary. Future research must strictly adhere to ARRIVE guidelines and standardize interventions to generate robust preclinical data, paving the way for high-quality clinical trials.

**Systematic Review Registration:**

https://www.crd.york.ac.uk/prospero/, identifier CRD4202459640.

## 1 Introduction

Type 2 diabetes mellitus (T2DM) is a chronic metabolic disorder characterized by persistent hyperglycemia resulting from insufficient insulin secretion and/or impaired insulin utilization (1, 2). It currently affects approximately 460 million people worldwide, and its prevalence continues to rise rapidly, projected to double within the next 25 years (3). Multi-system complications caused by T2DM have become a major cause of patient mortality (4). Sarcopenia has traditionally been regarded as an age-related geriatric syndrome. However, recent studies indicate that sarcopenia is not merely a consequence of aging, but rather a complex pathological condition involving bidirectional interactions with chronic metabolic diseases such as T2DM. Meta-analysis shows that the prevalence of sarcopenia among Asian T2DM patients is as high as 23% (5). Diabetic sarcopenia (DS) has been regarded as a new complication of diabetes, characterized by progressive reduction in skeletal muscle mass, decreased muscle strength and functional decline in diabetic patients (6, 7). This disease not only directly increases the risk of serious consequences such as fractures and limb disabilities but also establishes a two-way vicious cycle between sarcopenia and T2DM, mutually exacerbating disease progression (8, 9). DS has emerged as a significant public health concern that cannot be overlooked. However, current management strategies predominantly rely on non-specific approaches such as exercise intervention, nutritional support, and hormone replacement therapy (10). There remains a lack of specifically approved pharmacological treatments targeting this condition. Therefore, there is an urgent need to further explore effective prevention and treatment strategies for DS.

Traditional Chinese Medicine (TCM) has a long history in the prevention and treatment of diabetes and its complications, with core interventions encompassing herbal medicine, acupuncture, massage, and other therapeutic approaches (11). Within the theoretical framework of TCM, the pathological mechanism of DS is the same as the “Sanxiao” and “relaxation, weakness and muscle atrophy of limbs and tendons” as described under the syndrome of “diabetes with flaccidity” (12). In recent years, TCM researchers have conducted numerous beneficial explorations in the treatment of this condition. Through in-depth application of Chinese herbal medicine and other therapeutic methods, relatively promising efficacy has been achieved, and several related mechanisms have been preliminarily elucidated. However, current research in this field remains largely focused on animal experiments, and there is still a lack of sufficient clinical trial data to systematically evaluate the exact interventional effects of TCM on DS. Therefore, this study conducted a rigorous systematic review of literature on TCM interventions in DS animal models. It aims to scientifically evaluate the therapeutic effects of TCM and explore its potential mechanisms, thereby providing pre-clinical evidence to support future clinical trials.

## 2 Materials and methods

This systematic review complied with the Preferred Reporting Items for Systematic Examination and Meta-Analyses (PRISMA) guidelines (13). This study has been registered on the PROSPERO platform, with the registration number CRD42024596404.

### 2.1 Literature retrieval scheme

Two researchers independently and systematically searched multiple databases including PubMed, Web of Science, Cochrane, Embase, China National Knowledge Infrastructure (CNKI), VIP, and Wanfang. The initial literature search cutoff date was June 2024, with an updated search conducted in November 2025 during the manuscript revision phase. This covered all records from each database’s inception through November 2025. To minimize potential omissions, reference lists of relevant studies were also screened. Search terms included subject headings such as “diabetes mellitus, “ “sarcopenia, “ and “traditional Chinese medicine.” The specific search strategies for each database are detailed in **Supplementary Table S1**.

### 2.2 Studies selection

Inclusion criteria: ①*In vivo* studies in animals; ②Type 2 diabetes animal model was successfully built; ③The study set up an experimental group (receiving TCM treatment) and a control group (T2DM model group receiving placebo treatment or no administration); ④The study included at least one of the following outcomes: body weight, gastrocnemius muscle weight, grip strength, muscle cross-sectional area, muscle tissue morphology, blood glucose.

Exclusion criteria: ①Repeat published; ②Other kinds of studies (clinical trials, case reports, review articles, conference papers); ③Outcome data are insufficient.

Any disputes are resolved through discussion with a third reviewer.

### 2.3 Data extraction

Two reviewers independently conducted data abstraction, including the following information: characteristics of studies (the name of first author, publication year, study country); characteristics of the animal (species, sex, weight, sample size, modeling method); characteristics of intervention (intervention methods for the experimental and control groups; intervention dosage, route of administration, treatment duration, composition/acupoints, and method of preparing formulas for the experimental group); outcome index (body weight, gastrocnemius muscle weight, grip strength, muscle cross-sectional area, muscle tissue morphology, blood glucose).

For studies presenting data solely in graphical format without numerical text, numerical data extraction was performed using the Digitizer tool embedded in OriginPro software (Version 2021, OriginLab Corporation, Northampton, MA, USA). To ensure data reliability and minimize observer bias, data extraction was conducted independently by two reviewers. The results were cross-verified, and any discrepancies were resolved through discussion or by consulting a third reviewer to reach a consensus.

Regarding the handling of missing data, a strict protocol was followed. First, for studies reporting standard errors of the mean (SEM) or confidence intervals (CI), missing standard deviations (SD) were calculated using standard formulas recommended by the Cochrane Handbook. Second, for studies with undisclosed or ambiguous data (e.g., missing sample sizes or unclear grouping) that could not be derived calculationally, we attempted to contact the corresponding authors via email to request the raw data. However, no positive responses containing valid raw data were received. Consequently, to ensure the integrity of the results, studies with irretrievable critical data were excluded from the quantitative synthesis.

### 2.4 Quality assessment

Two reviewers independently assessed the quality of the included studies using the risk of bias tool from the Systematic Review Centre for Laboratory Animal Experimentation (SYRCLE) for animal studies (14). Based on the specific descriptions in each study, the risk of bias for each of the 10 items was assessed as “low”, “uncertain”, or “high” to reflect the quality of the study design and methodology.

### 2.5 Statistical analysis

Data statistical analysis was performed using Review Manager 5.3 software. The standardized mean difference (SMD) served as the measure of relative effect size. Effect size and its 95% confidence interval (CI) reflected the reliability of the results. Studies with low heterogeneity (P ≥ 0.1 or I² ≤ 50%) were analyzed using a fixed-effect model, while those with high heterogeneity employed a random-effects model.

## 3 Results

### 3.1 Study selection

**Figure 1:**
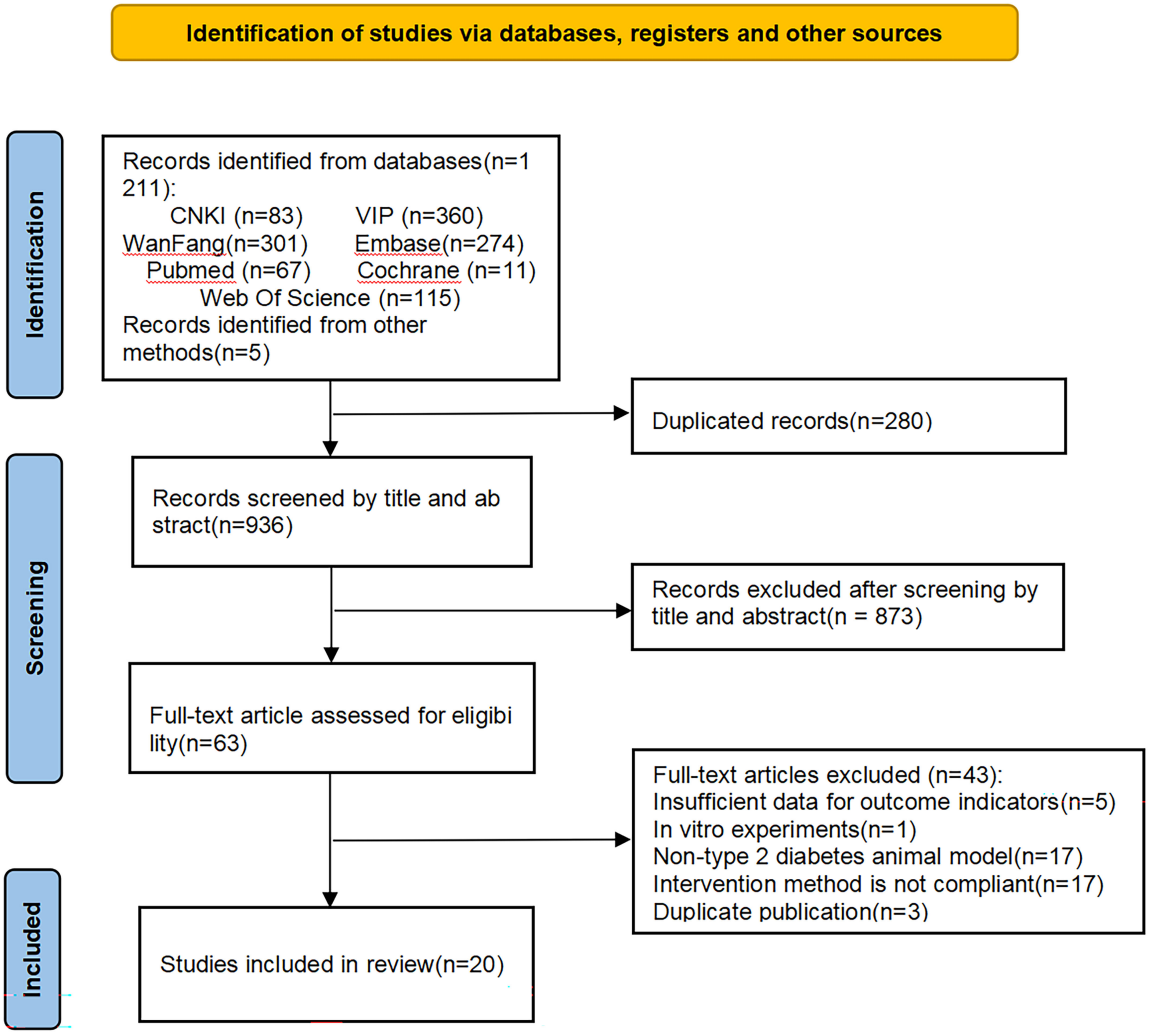
Flowchart of literature selection. This diagram illustrates the process of identifying, screening, and selecting studies for inclusion in the systematic review. Abbreviations: CNKI, China National Knowledge Infrastructure; VIP, Chinese Scientific Journals Database.

A systematic search of seven databases yielded 1, 216 relevant records. After plagiarism checking and the initial screening of the title and abstract, 1, 153 studies were excluded. The remaining 63 studies proceeded to full-text review. Using established selection criteria, 43 studies that did not meet requirements were further excluded, resulting in the inclusion of 20 studies (15–34). The detailed study selection process is outlined in **Figure 1**.

### 3.2 Characteristics of included studies

This study included 20 animal experiments involving a total of 351 animals. Among them, 177 animals were assigned to the experimental group and 174 to the control group. **Table 1** lists the main characteristics of the 20 included studies.

**Table 1:** Characteristics of studies included in the meta-analysis.

Study	Country	Animal (Sex)	Weight	Sample size		Model	Intervention							Outcome
							Method		Intervention details for the experimental group					
				EG	CG		EG	CG	Dose	Route of administration	Treatment duration	Composition / Acupoints	Method of preparing formulas	
She 2023 (15)	China	C57BL/6J mice(male)	20-28g	8	8	STZ	Astragulus embranaceus (Fisch.) Bge-Dioscorea opposita Thunb herb pair	Citrate buffer	7.8g·kg^−1^·d^−1^	Oral gavage	8 weeks	Astragulus embranaceus (Fisch.),Bge-Dioscorea opposita Thunb.	Aqueous extract	②④⑤
Tomoaki 2022 (16)	Japan	KKAy mice(male)	N/A	7	7	Spontaneous	Juzentaihoto	Blank	4% compound feed	Ad libitum feeding	8 weeks	Astragali Radix, Atractylodis Lanceae Rhizoma, Cinnamomi Cortex, Angelica Radix, Rehmanniae Radix, Ginseng Radix, Paeoniae Radix, Poria, Cnidii Rhizoma, Glycyrrhizae Radix.	Extract mixed in feed	①②④⑤⑥
Zhang 2014 (17)	China	C57BL/6J mice(male)	N/A	15	15	HFD+STZ	Zhimu-Huangbai Herb-Pair	Saline	2.6g·kg^−1^·d^−1^	Oral gavage	6 weeks	Anemarrhena, Phellodendron.	Ethanol Extract	①②③④⑤⑥
Guo 2024 (18)	China	Wistar rats(male)	220-240g	8	8	HFD+STZ	Massage	Blank	Vibration and Point-pressing technique(approx. 26 min/session, 1/day)	Surface massage	6 weeks	Vibration technique applied to Shenque and bilateral muscles including the biceps brachii, triceps brachii, quadriceps femoris, hamstrings, and gastrocnemius; Point-pressing technique applied to bilateral Yishu, Pishu, Shenshu, Shousanli, Fenglong, Xuehai, Chengshan, Zusanli, Sanyinjiao, Quchi, and Hegu.	N/A	②③⑤
Meng 2023 (19)	China	Wistar rats(male)	200-220g	6	6	HFD+STZ	Massage	Blank	Dian and Rou techniques(15 min/session, 1/day)	Surface massage	8 weeks	Acupoints: Fenglong, Zusanli, Sanyinjiao, Xuehai; Muscle regions: bilateral gastrocnemius and quadriceps femoris.	N/A	①②③⑥
Zhao 2024a (20)	China	ZDF(fa/fa) rats(male)	N/A	6	6	Spontaneous	Dahuang Tangluo Pill	Saline	2.16g·kg^−1^·d^−1^	Oral gavage	12 weeks	Rheum palmatum, Coptis chinensis, Scutellaria baicalensis, Zingiber officinale, Pueraria lobata, Panax notoginseng, Panax quinquefolius.	Finished Products	①⑤⑥
Sun 2025 (21)	China	Wistar rats(male)	N/A	6	6	HFD+STZ	Massage	Blank	Dian and Zhen techniques(34 min/session, 1/day)	Surface massage	8 weeks	Dian technique applied to Huantiao, Xuehai, Zusanli, Fenglong, Sanyinjiao, Chengshan; Zhen technique applied to Shenque, Zhongwan, Tianshu, Liangmen, Qihai.	N/A	⑤⑥
Wang 2024 (22)	China	C57BL/6J mice(male)	19-23g	11	11	HFD+STZ	Buyang Huanwu tang	Saline	86.5g·kg^−1^·d^−1^	Oral gavage	8 weeks	Astragalus membranaceus, Angelica sinensis, Paeonia lactiflora, Pheretima aspergillum, Ligusticum chuanxiong, Carthamus tinctorius, Prunus persica.	Aqueous extract	①⑤⑥
Zhu 2015 (23)	China	KKAy mice(male)	N/A	14	14	Spontaneous	Shenqi Compound	Saline	14.4g·kg^−1^·d^−1^	Oral gavage	8 weeks	Rehmannia glutinosa, Dioscorea opposita, Cornus officinalis, Astragalus membranaceus, Panax ginseng, Salvia miltiorrhiza, prepared Rheum palmatum, Trichosanthes kirilowii.	Finished Products	⑤⑥
Ma 2024 (24)	China	SD rats(male)	160-200g	8	8	HFD+STZ	Total Astragalus saponins	Saline	80mg·kg^−1^·d^−1^	Oral gavage	12 weeks	N/A	Finished product	②⑤⑥
Zhao 2024b (25)	China	SD rats(male)	140-180g	6	6	HFD+STZ	Qinlian Jiangxia Decoction	Blank	3.465g·kg^−1^·d^−1^	Oral gavage	12 weeks	Prepared Pinellia ternata, Scutellaria baicalensis, Coptis chinensis, dried Zingiber officinale.	Finished Products	①②④⑤⑥
Zhong 2018 (26)	China	GK rats(male)	271-338g	10	9	Spontaneous	Shenqi compound	Saline	1.44g·kg^−1^·d^−1^	Oral gavage	8 weeks	Panax ginseng, Astragalus membranaceus, Salvia miltiorrhiza, Dioscorea opposita, prepared Rheum palmatum, Rehmannia glutinosa, Trichosanthes kirilowii, Cornus officinalis.	Aqueous extract	①②⑤⑥
Zhong 2025 (27)	China	Wistar rats(male)	200-220g	5	6	HFD+STZ	Shenqi compound	Saline	1.44g·kg^−1^·d^−1^	Oral gavage	8 weeks	Panax ginseng, Astragalus membranaceus, Salvia miltiorrhiza, Dioscorea opposita, prepared Rheum palmatum, Rehmannia glutinosa, Trichosanthes kirilowii, Cornus officinalis.	Finished product	④⑤
Zuo 2022 (28)	China	SD rats(male)	N/A	10	10	HFD+STZ	Campanumoea javanica Bl	Saline	6.3g·kg^−1^·d^−1^	Oral gavage	4 weeks	N/A	Aqueous extract	①②③⑤⑥
Xv 2022 (29)	China	C57BL/6J mice(male)	>30g	16	16	HFD+STZ	Osteoking	Saline	0.59ml·kg^−1^(Every other day)	Oral gavage	12 weeks	Panax notoginseng, Astragalus membranaceus, Panax ginseng, Carthamus tinctorius, Eucommia ulmoides, Citrus reticulata, etc.	Finished product	①②③④
Huang 2011 (30)	China	Wistar rats(male)	160-180g	8	8	STZ	Mulberry leaf polysaccharide	Distilled water	4ml·d^−1^	Oral gavage	6 weeks	N/A	Finished product	①⑥
Shi 2023 (31)	China	Wistar rats(male)	200-220 g	9	9	HFD+STZ	Mulberry leaf extract	Distilled water	4.0g·kg^−1^·d^−1^	Oral gavage	8 weeks	N/A	Aqueous extract	①⑤⑥
Qi 2017 (32)	China	Wistar rats(male)	200-240g	12	9	HFD+STZ	Jianpi Fang	Pure water	20g·kg^−1^·d^−1^	Oral gavage	6 weeks	Raw Astragalus membranaceus, Dioscorea opposita, Pseudostellaria heterophylla, Poria cocos, Atractylodes macrocephala, Coptis chinensis, Citrus reticulata, stir-baked Endothelium Corneum Gigeriae Galli, Polygonatum sibiricum, Pueraria lobata, Ophiopogon japonicus, Salvia miltiorrhiza, Panax notoginseng powder.	Aqueous extract	①⑤⑥
Wang 2021 (33)	South Korea	C57BL/6N mice(male)	NR	5	5	HFD+STZ	Root extract of Morinda officinalis	Blank	200 mg·kg^−1^·d^−1^	Oral gavage	4 weeks	N/A	Aqueous extract	①④⑤⑥
Ou 2022 (34)	China	KKAy mice(male)	NR	7	7	Spontaneous	Saikokeishikankyoto	Blank	4% compound feed	Ad libitum feeding	6 weeks	Bupleuri Radix、Scutellariae Radix、Cinnamomi Cortex、Trichosanthis Radix、Ostreae Testa 、Zingiberis Rhizoma、Glycyrrhizae Radix	Extract mixed in feed	①②④⑥

EG, experimental group; CG, control group; NR, Not reported; N/A, not applicable; STZ, Streptozotocin; HFD+STZ, High-Fat Diet+Streptozotocin; KKAy mice, GK rats and ZDF(fa/fa) rats serve as hereditary diabetes models, exhibiting a diabetic phenotype derived from genetic mutations without requiring exogenous induction; Blank: No intervention measures were implemented. Outcomes: ① Body weight; ② Gastrocnemius muscle weight; ③ Grip strength; ④ Muscle cross-sectional area; ⑤ Muscle tissue morphology; ⑥ Blood glucose.

### 3.3 Quality assessment

**Figure 2:**
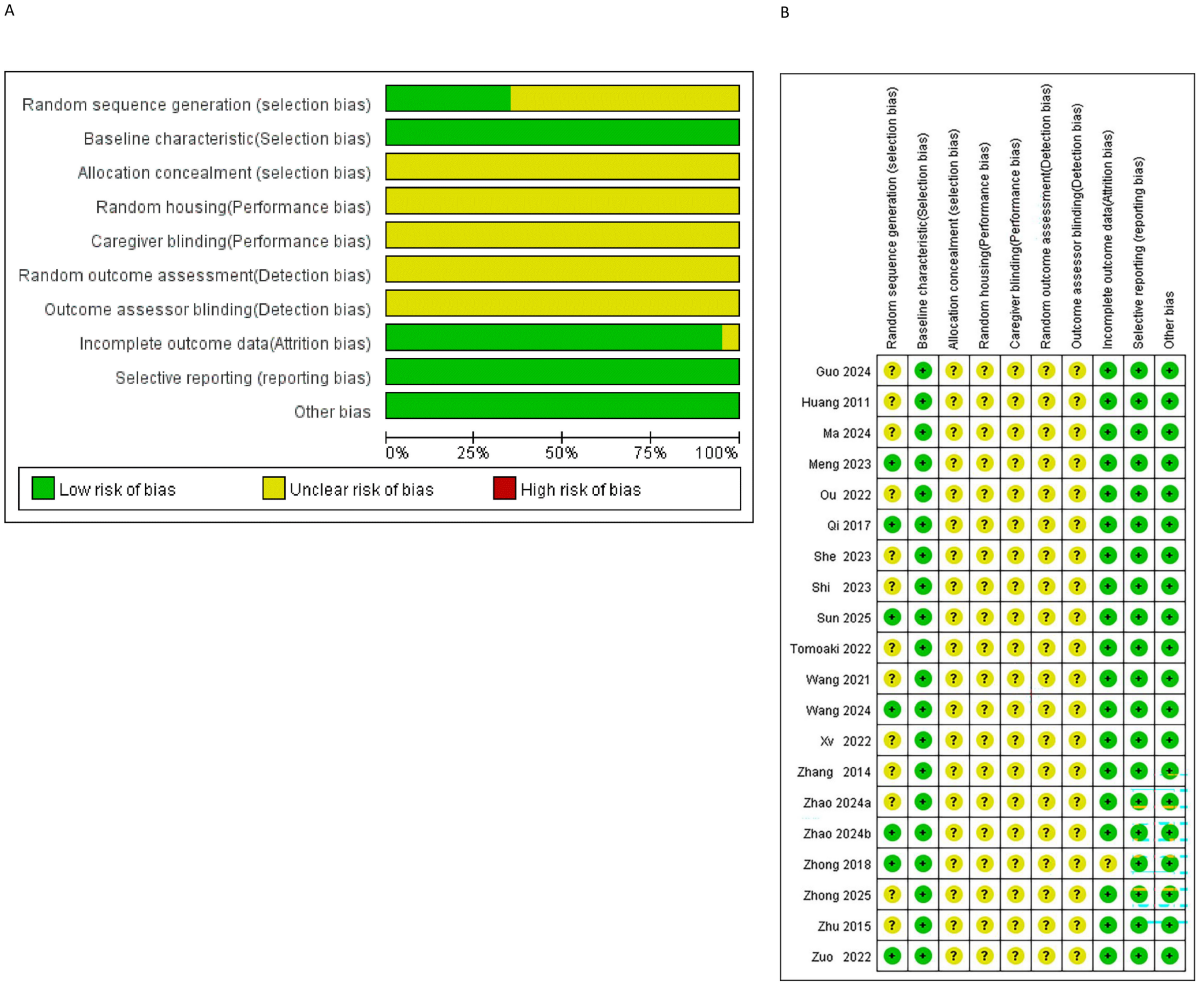
Risk of bias assessment results. **(A)** Risk of bias graph. **(B)** Risk of bias summary.

Based on the SYRCLE bias risk assessment tool, the 20 included studies were evaluated, with specific results shown in **Figure 2A** and **2B**. Regarding random sequence generation, 7 studies employed random number tables, indicating a low risk of selection bias (19, 21, 22, 25, 26, 28, 32). The remaining 13 studies mentioned only “randomization” without specifying the method, resulting in an unclear risk of selection bias (15–18, 20, 23, 24, 27, 29–31, 33, 34). Regarding baseline characteristics, all studies demonstrated consistency in baseline traits such as strain, sex, and age among experimental animals. No significant differences were observed in core indicators (e.g., body weight, blood glucose) between groups after randomization, indicating a low risk of selection bias. For allocation concealment, none of the studies mentioned whether the allocation sequence was concealed from the personnel, resulting in an unclear risk of selection bias. Regarding random housing and blinding of personnel, none of the studies reported randomization of animal placement or blinding of animal caretakers and medication administrators, leaving the risk of performance bias unclear. For random outcome assessment and blinding of outcome assessors, none explicitly stated whether animals underwent outcome measurements in randomized order or whether assessors were blinded, rendering the risk of detection bias unclear. Regarding incomplete outcome data, two studies reported animal withdrawals. One study did not specify the reasons for withdrawal, resulting in an unclear risk of attrition bias (26). The other study comprehensively reported withdrawals with no apparent abnormalities in distribution across groups, indicating a low risk of attrition bias (32). The remaining 18 studies reported no animal withdrawals and had complete data, resulting in a low risk of attrition bias (15–25, 27–31, 33, 34). Regarding selective reporting, no studies showed clear signs of selective reporting, indicating a low risk of reporting bias. For other biases, no other significant sources of bias were identified, resulting in a low risk of other biases.

### 3.4 Primary outcomes

#### 3.4.1 Body weight

**Figure 3:**
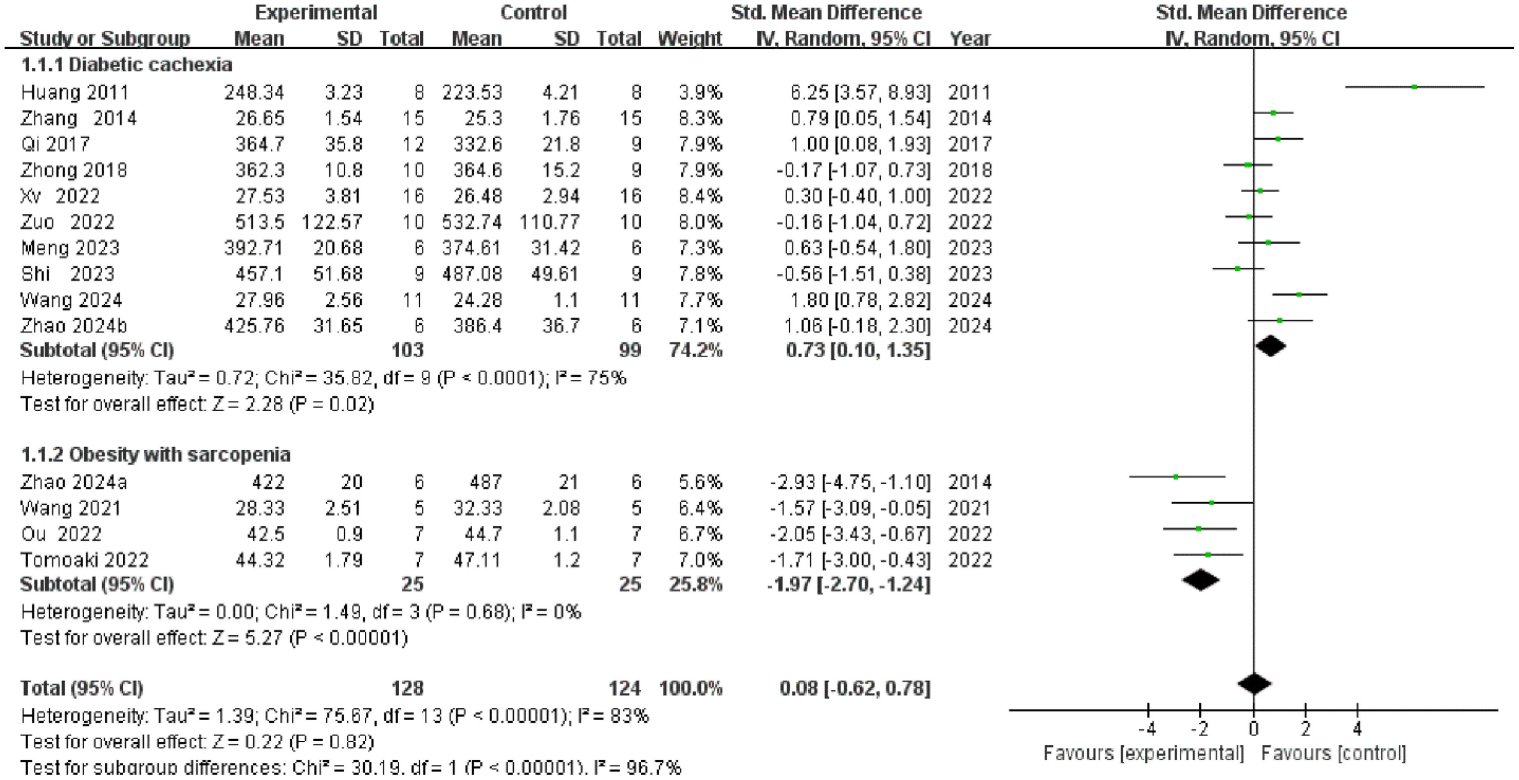
Forest plot of body weight.

A total of 14 studies reported weight measurements (**Figure 3**). The overall meta-analysis indicated no statistically significant effect of TCM interventions on body weight [SMD = 0.08, 95% CI (-0.62, 0.78), P = 0.82], with high heterogeneity (I² = 83%). Subgroup analysis (by model type) revealed significant inter-group differences (P < 0.00001), indicating model type as a major source of heterogeneity. Within the diabetes cachexia model subgroup, intervention significantly increased body weight [SMD = 0.73, 95% CI (0.10, 1.35), P = 0.02]; In the obesity with sarcopenia model subgroup, the intervention significantly reduced body weight [SMD = −1.97, 95% CI (−2.70, −1.24), P < 0.00001], with no heterogeneity within this subgroup (I² = 0%). This finding indicates that traditional Chinese medicine exerts a bidirectional regulatory effect on body weight, rather than being ineffective.

#### 3.4.2 Gastrocnemius muscle weight

**Figure 4:**
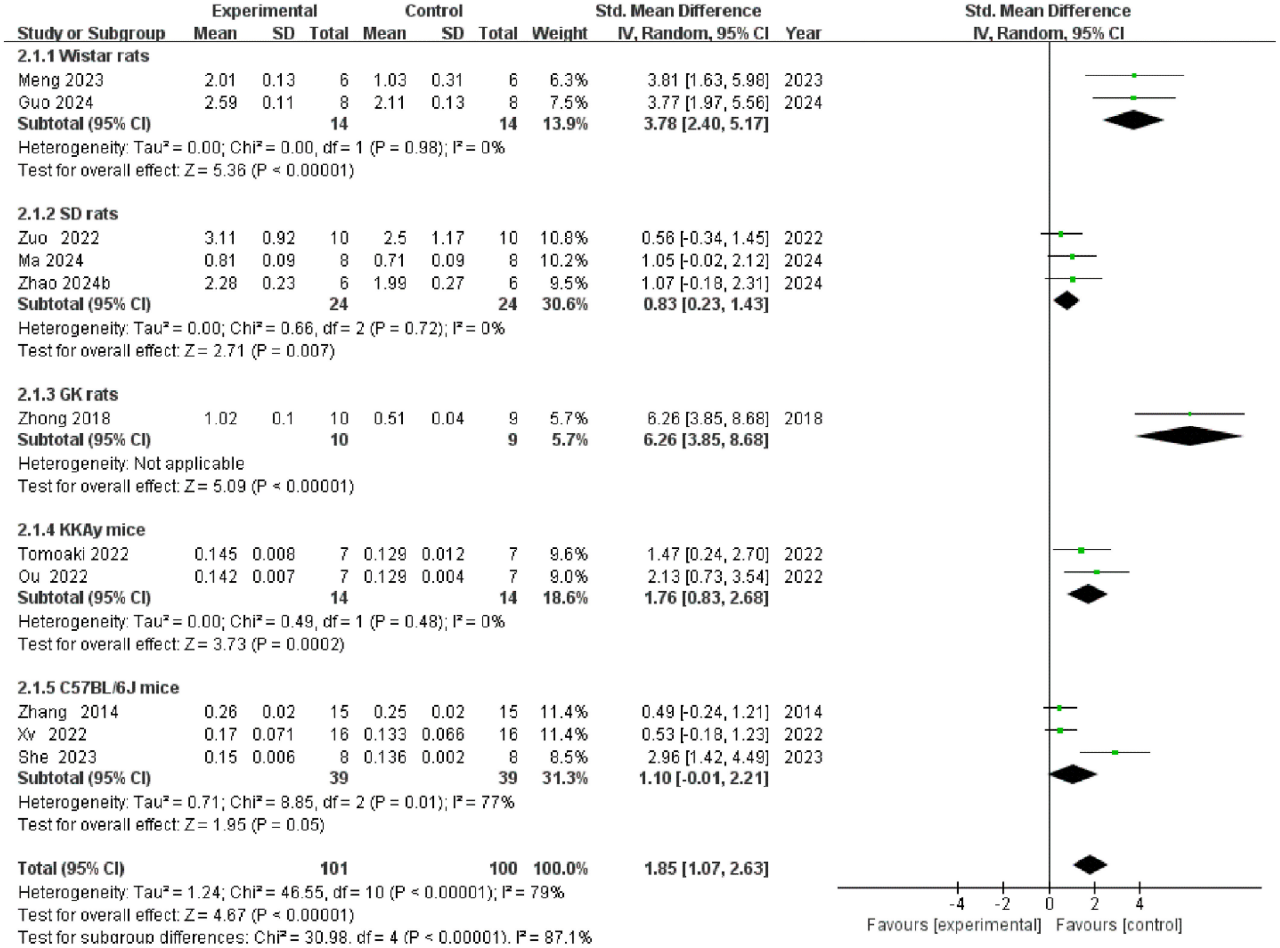
Forest plot of gastrocnemius muscle weight.

A total of 11 studies reported gastrocnemius muscle weight (**Figure 4**). The overall meta-analysis demonstrated that TCM interventions significantly increased gastrocnemius muscle weight [SMD = 1.85, 95% CI (1.07, 2.63), P < 0.00001], albeit with high heterogeneity (I² = 79%). Subgroup analysis stratified by animal strain revealed significant between-group differences (P < 0.00001), identifying animal strain as a major source of heterogeneity. The intervention significantly increased gastrocnemius muscle weight in the Wistar rat, SD rat, GK rat, and KKAy mouse subgroups, with heterogeneity being absent or not applicable in these groups (I² = 0% or N/A). In contrast, in the C57BL/6J mouse subgroup, the intervention effect was not statistically significant [SMD = 1.10, 95% CI (-0.01, 2.21), P = 0.05], and considerable heterogeneity persisted (I² = 77%).

#### 3.4.3 Grip strength

**Figure 5:**
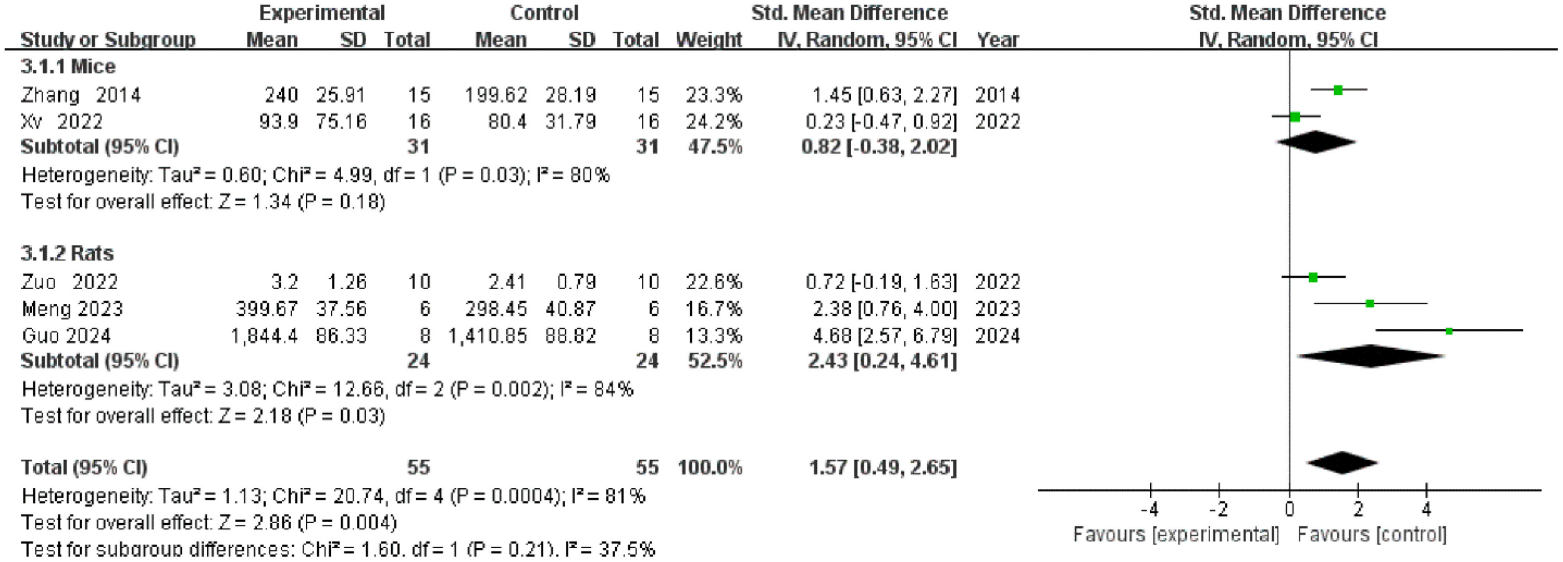
Forest plot of grip strength.

A total of 5 studies reported grip strength data (**Figure 5**). The overall meta-analysis indicated that TCM intervention significantly increased grip strength compared to the control group [SMD = 1.57, 95% CI (0.49, 2.65), P = 0.004], although substantial heterogeneity was observed (I² = 81%). Subgroup analysis based on animal species (mice vs. rats) revealed no statistically significant difference between groups (P = 0.21), suggesting that species may not be the primary source of heterogeneity. Specifically, while the intervention significantly improved grip strength in the rat subgroup [SMD = 2.43, 95% CI (0.24, 4.61), P = 0.03], it failed to achieve statistical significance in the mouse subgroup [SMD = 0.82, 95% CI (-0.38, 2.02), P = 0.18], where heterogeneity remained high (I² = 80%).

#### 3.4.4 Muscle cross-sectional area

**Figure 6:**
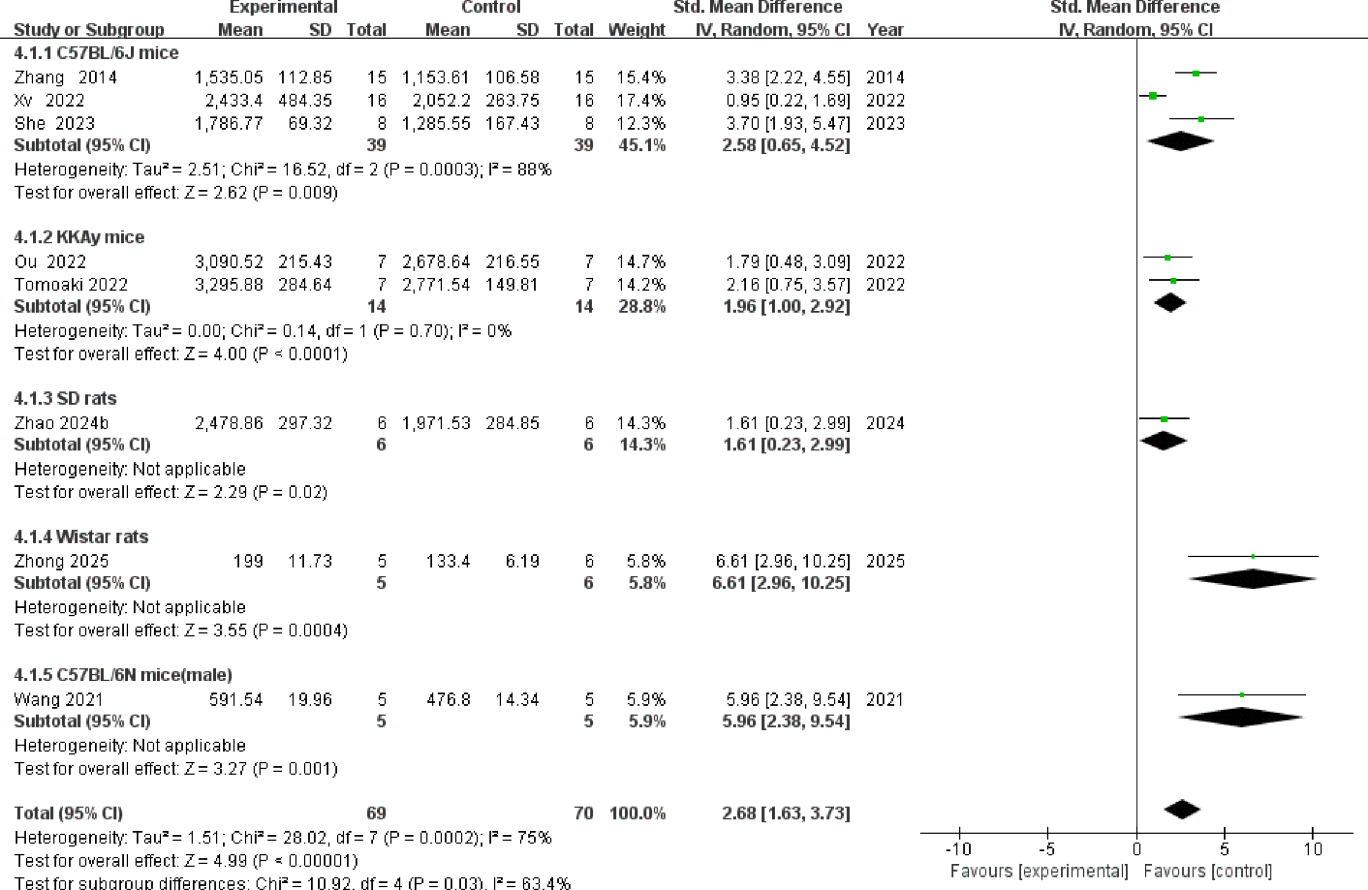
Forest plot of muscle cross-sectional area.

A total of 8 studies reported muscle cross-sectional area (**Figure 6**). The overall meta-analysis showed that TCM intervention significantly increased muscle cross-sectional area [SMD = 2.68, 95% CI (1.63, 3.73), P < 0.00001], despite the presence of substantial heterogeneity (I² = 75%). Subgroup analysis stratified by animal strain revealed a statistically significant difference between groups (P = 0.03), indicating that animal strain is a significant source of heterogeneity. Specifically, the intervention consistently and significantly increased muscle cross-sectional area across the C57BL/6J, KKAy, SD, Wistar, and C57BL/6N subgroups. Notably, the Wistar rat subgroup exhibited the largest effect size [SMD = 6.61, 95% CI (2.96, 10.25), P = 0.0004], whereas high heterogeneity persisted within the C57BL/6J mouse subgroup (I² = 88%).

#### 3.4.5 Muscle tissue morphology

A total of 16 studies utilized H&E staining to compare muscle tissue morphology between the experimental and control groups, and a qualitative analysis was performed (**Table 2**). The results indicated that muscle morphology in the experimental group was improved compared to the control group. To more objectively evaluate the impact of TCM on muscle tissue in diabetic animals, a supplementary quantitative micromorphological analysis was conducted for studies that provided quantitative data. The results suggest that TCM plays a positive role in the restoration of muscle pathological morphology in animals with type 2 diabetes mellitus.

**Table 2:** Qualitative and quantitative assessment of muscle tissue morphology.

Study	H&E staining results		Quantitative Comparison of Microstructural Features		
	EG	CG	Indicator Type	Numerical value	P-value
She 2023 (15)	The arrangement of muscle fibers was significantly more regular compared to the aged type 2 diabetes model group; the width of intermuscular spaces recovered to levels close to the normal aged group, with inflammatory cell infiltration largely resolved; striation clarity improved, and the distribution of muscle cell nuclei tended toward normalcy.	Muscle fibers show disorganized arrangement with extensive atrophy; intermuscular spaces are markedly widened, revealing sparse lymphocytic infiltration; striations are blurred or absent, and muscle cell nuclei exhibit condensation and displacement.	CSA	EG: 1786.77±69.32 CG: 1285.55±167.43	P < 0.05
Tomoaki 2022 (16)	Muscle fiber atrophy is reduced, muscle gaps are narrowed, and striated structures are more clearly defined.	Muscle fiber diameter reduction, disordered arrangement, and increased intermuscular space.	CSA	EG: 3295.88±284.6 CG: 2771.54±149.81	P < 0.05
Zhang 2014 (17)	Improved leftward shift in muscle fiber size distribution.	The distribution of muscle fiber sizes also shifted to the left.	CSA	EG: 1535.05±112.9 CG: 1153.61±106.58	P < 0.05
Guo 2024 (18)	Muscle cells are arranged more tightly, muscle fiber atrophy is reduced, and the number of inflammatory cells is decreased.	Muscle cells are disorganized and loosely arranged, with muscle fibers showing marked atrophy and thinning, accompanied by significant inflammatory cell infiltration.	N/A		
Zhao 2024a (20)	The morphological structure of skeletal muscle cells is basically intact, with a reduction in nuclear aggregation and displacement. The muscle cells are closely arranged, and the proliferation of intercellular Spaces and connective tissue is decreased, as well as the infiltration of inflammatory cells and vacuolated lipid droplets.	Cross-sectional cells show angular atrophy with scattered necrosis. Cell membranes exhibit blurred borders, while nuclei appear aggregated, reduced in number, and displaced toward the cell periphery. Muscle cells are disorganized and loosely arranged, with widened intercellular spaces. Significant connective tissue proliferation, inflammatory cell infiltration, and vacuolar lipid droplets are present.	N/A		
Sun 2025 (21)	Muscle tissue is neatly arranged, showing focal areas of muscle cell atrophy. Muscle cell edema is present but mild in severity and limited in extent, accompanied by a small number of inflammatory cells.	Muscle tissue arrangement is disordered, muscle fibers are reduced in size, muscle cells are atrophied and edematous, muscle interstices are enlarged, and there is minimal autolysis of muscle fibers.	N/A		
Wang 2024 (22)	Pathological conditions such as muscle fiber atrophy in skeletal muscle cells showed significant improvement, with partial restoration of normal fiber bundle morphology.	Skeletal muscle fibers show generalized atrophic thinning, enlarged and irregular intercellular spaces with increased connective tissue, and focal degradation and loss of myofibrils, indicating skeletal muscle atrophy and morphological structural alterations.	N/A		
Zhu 2015 (23)	Muscle tissue is arranged in a regular pattern with uniformly sized muscle cells. Scattered foci of muscle cell atrophy are visible. Areas of muscle cell edema are small and mild in severity. There is mild inflammatory cell infiltration. Muscle cell necrosis and fragmentation are uncommon.	Muscle tissue arrangement is disorganized, with marked atrophy and reduced cell volume. Extensive cellular edema is present, accompanied by significant inflammatory cell infiltration. Large areas of focal muscle tissue disruption are observed, without evidence of prominent fibrous connective tissue proliferation.	N/A		
Ma 2024 (24)	The arrangement of muscle fibers is more orderly than in the model group, but shows a certain degree of looseness. Cell morphology is largely intact, with relatively regular cell arrangement and narrowed intercellular spaces.	Skeletal muscle fibers exhibit disorganized and loose arrangement, with enlarged gaps between myocytes and a marked reduction in cross-sectional area. Myocytes show atrophy, and muscle fibers display minor autolysis.	N/A		
Zhao 2024b (25)	The morphology and structure of the gastrocnemius muscle are essentially normal.	The morphology and structure of the gastrocnemius muscle are essentially normal.	CSA	EG: 2478.86±297.3 CG: 1971.53±284.85	P < 0.01
Zhong 2018 (26)	Skeletal muscle tissue is neatly arranged, showing focal areas of muscle cell atrophy. Muscle cell edema is present but mild in severity and limited in extent, accompanied by minimal inflammatory cell infiltration. Occasional muscle cell disruption and necrosis are observed.	Muscle tissue arrangement is disorganized, with muscle cells showing atrophy and edema. Inflammatory cell infiltration is prominent, accompanied by minor autolysis of muscle fibers and mild disruption of the fascicular membranes.	N/A		
Zhong 2025 (27)	Only mild inflammatory cell infiltration and fibrous proliferation are observed.	The structure of the light and dark bands in muscle fibers is indistinct, and muscle cells are irregularly arranged. Inflammatory cell infiltration is observed in the interstitial muscle tissue, with no evidence of fibrotic deposition.	CSA	EG: 199±11.73 CG: 133.4±6.19	P < 0.05
Zuo 2022 (28)	Cellular edema has subsided, with a partial recovery in the number of nuclei and muscle fibers.	Disordered arrangement of gastrocnemius muscle fibers, cellular atrophy, edema, inflammatory cell infiltration, partial autolysis of muscle fibers, and mild rupture of the sarcolemma.	N/A		
Shi 2023 (31)	Skeletal muscle fibers are neatly arranged, with reduced interstitial spaces and atrophy.	Mild atrophy with mild eosinophilic degeneration of muscle fibers and widened interstices.	Histology score of skeletal muscle	EG: 0.96±1.01 CG: 1.97±0.97	P < 0.01
Qi 2017 (32)	Cells exhibit regular morphology, orderly arrangement, and well-defined borders.	Cell morphology is disorganized, with enlarged intercellular spaces and blurred boundaries.	N/A		
Wang 2021 (33)	Muscle fibres are closely arranged, with structural recovery approaching normal levels.	The muscle fibre bundles are more widely separated from one another; there are distinct gaps between the bundles; the muscle fibres are often circular to angular in shape and loosely arranged.	CSA	EG: 591.54±19.96 CG: 476.8±14.34	P<0.001

EG, experimental group; CG, control group; N/A, not applicable; CSA: Cross-Sectional Area (units: μm²); Values are presented as mean ± standard deviation; P-values are derived from comparative statistical analyses between EG and CG; H&E staining was used for qualitative assessment of muscle morphology.

#### 3.4.6 Blood glucose

**Figure 7:**
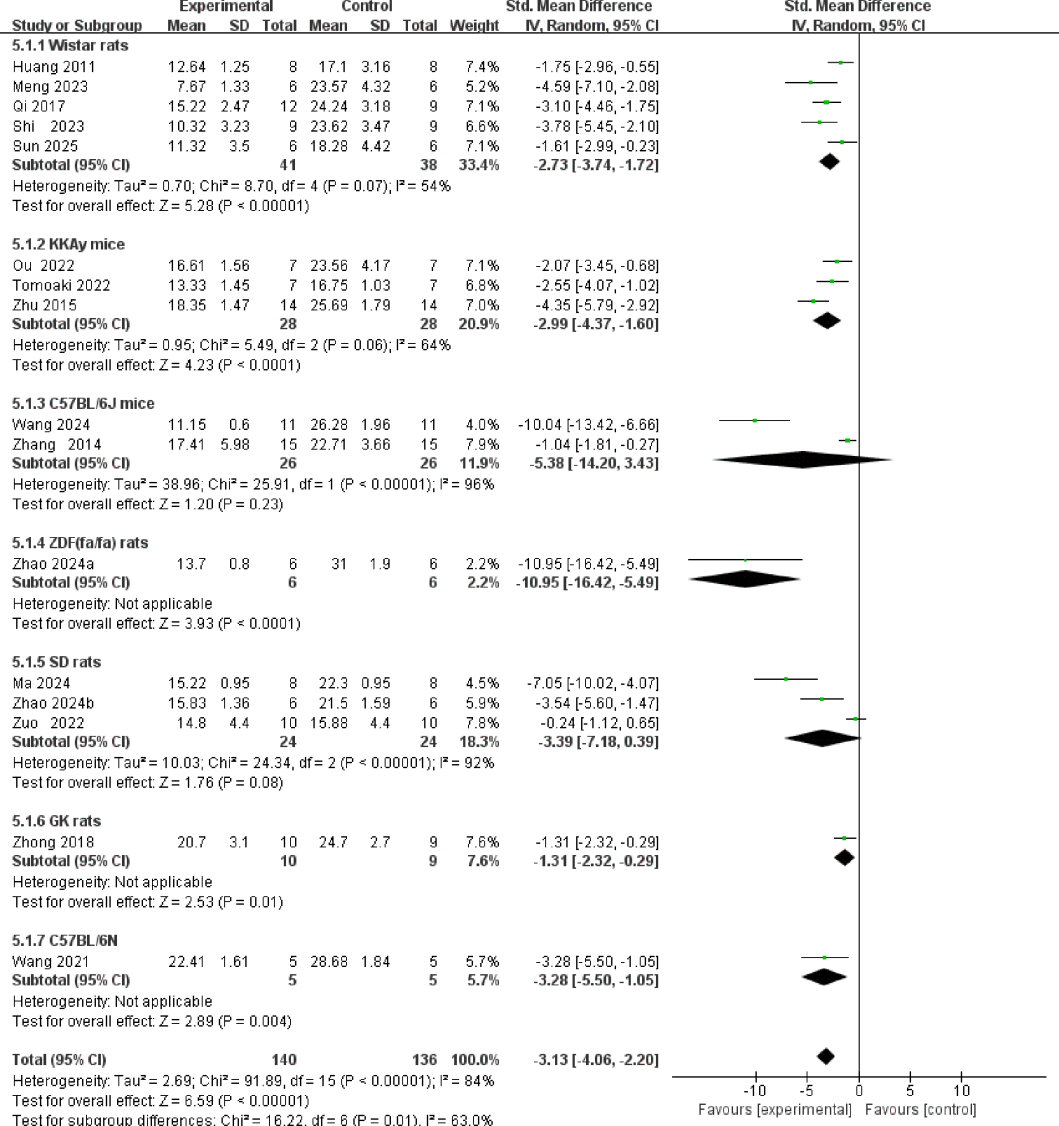
Forest plot of blood glucose.

A total of 16 studies reported blood glucose levels (**Figure 7**). The overall meta-analysis indicated that TCM intervention significantly reduced blood glucose levels [SMD = -3.13, 95% CI (-4.06, -2.20), P < 0.00001], although high heterogeneity existed among the studies (I² = 84%). Subgroup analysis stratified by animal strain revealed statistically significant differences between groups (P = 0.01), suggesting that animal strain is a potential source of heterogeneity. The intervention demonstrated significant hypoglycemic effects in the Wistar rat, KKAy mouse, ZDF(fa/fa) rat, GK rat, and C57BL/6N mouse subgroups. However, in the C57BL/6J mouse subgroup, although the effect size was negative, it did not reach statistical significance [SMD = -5.38, 95% CI (-14.20, 3.43), P = 0.23], and heterogeneity within this subgroup was extremely high (I² = 96%). Similarly, the SD rat subgroup failed to show statistical significance [SMD = -3.39, 95% CI (-7.18, 0.39), P = 0.08], with consistently high heterogeneity (I² = 92%).

### 3.5 Regression analysis

Given the limited number of studies included for each outcome measure (N = 5–16), only univariate meta-regression was employed to explore sources of heterogeneity (multivariate models were not utilized due to insufficient statistical power). Although most variables did not reach statistical significance (P < 0.05), certain covariates demonstrated strong explanatory power regarding heterogeneity. For body weight, pathological state was the primary source of heterogeneity (P = 0.076), accounting for 25.41% of the between-study variation. Regarding gastrocnemius muscle weight, intervention type (P = 0.103) and disease model (P = 0.196) explained 25.44% and 13.35% of the heterogeneity, respectively. For grip strength, although animal species did not reach statistical significance (P = 0.142), it explained 52.67% of the heterogeneity. Regarding muscle cross-sectional area, treatment duration showed a marginal association with effect size (P = 0.158), explaining 40.14% of the heterogeneity. For blood glucose, treatment duration exhibited the highest explanatory power (Adj R² = 24.66%, P = 0.105). Overall, while some covariates explained a portion of the heterogeneity, residual heterogeneity remained high across all models (I²_res > 66%), suggesting the influence of other unaccounted factors. Detailed univariate regression values for each outcome are presented in **Supplementary Table S2**.

### 3.6 Sensitivity analysis

Sensitivity analyses were conducted using the leave-one-out method and by restricting inclusion to studies that explicitly reported randomization methods to verify the robustness of the results. The leave-one-out analysis revealed that the pooled effect sizes for muscle cross-sectional area, gastrocnemius muscle weight, body weight, and blood glucose did not exhibit any directional changes after excluding any single study, indicating the robustness of the results. Although the grip strength index showed some fluctuation, the overall conclusion remained unreversed.

When restricted to studies explicitly reporting randomization methods, the results for gastrocnemius muscle weight and blood glucose were largely consistent with the main analysis, demonstrating good robustness. Notably, for body weight, heterogeneity significantly declined after excluding low-quality studies (I² decreased from 83% to 59%), and the effect approached statistical significance (P = 0.05), suggesting that study quality is a major source of heterogeneity for this outcome. In contrast, grip strength failed to reach statistical significance after restricting by study quality (P = 0.09), indicating weaker robustness and requiring cautious interpretation. Analysis was not performed for muscle cross-sectional area as only one study explicitly reported the randomization method. For specific data, please refer to **Supplementary Table S3** and **Supplementary Figures S1**, **S2**.

### 3.7 Publication bias

The analysis of publication bias revealed variations across different outcome measures. For body weight, Egger’s test detected no significant publication bias (P = 0.979), and the funnel plot was largely symmetrical. In contrast, significant publication bias was observed for gastrocnemius muscle weight (P < 0.001); after adjustment using the trim-and-fill method, two studies were imputed, reducing the effect size from 2.004 to 1.526, which suggests that the original results may have overestimated the intervention efficacy. Although grip strength (P = 0.033), muscle cross-sectional area (P = 0.005), and blood glucose (P < 0.001) all exhibited evidence of small-study effects, the trim-and-fill analysis showed that effect sizes remained stable before and after imputation (grip strength: 1.702; muscle cross-sectional area: 2.932; blood glucose: -3.384). This indicates that the current body of evidence is relatively complete, and the pooled results are reliable. Nevertheless, caution should be exercised when interpreting the results for gastrocnemius muscle weight and grip strength, considering the influence of potential bias. For specific data, please refer to **Supplementary Table S4** and **Supplementary Figures S3**, **S4**.

## 4 Discussion

### 4.1 Summary of main findings

This is the first systematic review and meta-analysis that has evaluated the therapeutic efficacy of TCM interventions in DS animal models. Results suggest that specific TCM interventions may significantly improve core pathological features of DS, including increasing gastrocnemius muscle weight and cross-sectional area, enhancing muscle tissue morphology, and improving grip strength. Additionally, the analysis indicates significant hypoglycemic effects in treated groups. Notably, a potential bidirectional regulatory effect on body weight was observed across different models: interventions appeared to promote weight gain in diabetic cachexia models whilst inducing weight loss in obesity with sarcopenia models. This bidirectional phenomenon may suggest that the metabolic impact of these TCM treatments is context-dependent, offering potential theoretical contributions to personalized diabetes management. Although the outcome measures exhibited considerable heterogeneity in the overall analysis, subgroup analyses and meta-regression identified ‘pathological state’ and ‘animal species’ as key factors contributing to therapeutic variability.

### 4.2 Potential molecular mechanisms of TCM in DS

The development of DS is a complex pathological process involving multifactorial, multi-pathway interactions, whose essence appears to lie in the vicious cycle formed by systemic metabolic disorders and dysregulation of intramuscular molecular networks. The core pathophysiological alteration in this disease is thought to be the imbalance in skeletal muscle protein turnover homeostasis—specifically, the suppression of protein synthesis coupled with the overactivation of degradation pathways, which may ultimately lead to net muscle protein loss (35). This protein turnover imbalance is closely associated with key pathological mechanisms including insulin resistance (IR), chronic inflammation, oxidative stress, and mitochondrial damage. These mechanisms likely interact synergistically, collectively driving the pathological progression of skeletal muscle mass reduction, strength decline, and functional deterioration. This study systematically reviewed the potential mechanisms of various TCM intervention in DS, encompassing 20 studies (see **Supplementary Material Table S5**). These proposed mechanisms observed across different preclinical models can be broadly categorized into three aspects:

#### 4.2.1 Direct regulation of protein turnover homeostasis

Under physiological conditions, skeletal muscle protein balance is maintained through the coordinated regulation of proteolytic and protein synthesis pathways. The major proteolytic pathways (including the ubiquitin-proteasome system, lysosomal autophagy, and the caspase and calpain systems) are regulated by signaling pathways such as Interleukin-6/Signal Transducer and Activator of Transcription (IL6/STAT), Tumor Necrosis Factor-alpha/Nuclear Factor Kappa B and Interleukin-6/NF-κB(TNF&IL6/NFκB), Myostatin/Mothers Against Decapentaplegic Homolog 2/3 (myostatin/Smad2/3), and Forkhead box O1/3 (FoxO1/3) signaling pathways. Conversely, protein synthesis is primarily regulated by the Insulin-like Growth Factor 1–Phosphoinositide 3-Kinase–Protein Kinase B–Mammalian Target of Rapamycin (IGF1-PI3K-Akt-mTOR) and Stem Cell - G Protein Subunit Alpha i2 (SC-Gαi2) signaling pathways (36, 37). These pathways also represent common therapeutic targets for DS. Based on the reviewed literature, the mechanisms of specific TCM treatments for DS appear to involve the modulation of the IGF1-PI3K-AKT-mTOR protein synthesis pathway and the FoxO-mediated protein degradation pathway, depending on the formulation and experimental context.

The IGF1-PI3K-AKT-mTOR pathway is the primary pathway for skeletal muscle protein synthesis. Within the IGF1-PI3K-AKT-mTOR pathway, IGF1 and its downstream intracellular signaling molecules play a crucial role in regulating skeletal muscle growth (38). IGF serves as a positive regulatory signal for muscle growth, thereby promoting the proliferation, differentiation, and fusion of skeletal muscle stem cells into myotubes (39). PI3K-Akt constitutes the downstream signaling pathway of IGF-1, which induces muscle mass increase by stimulating this pathway (40, 41). Akt further activates mTOR, a protein kinase in the PI3K-related kinase family that is the catalytic subunit of two distinct complexes: mTORC1 and mTORC2. mTORC1 regulates protein synthesis by phosphorylating Ribosomal Protein S6 Kinase Beta-1 (S6K1) and Eukaryotic Translation Initiation Factor 4E-Binding Protein 1 (4EBP1) (42). Phosphorylated S6K1 and phosphorylated 4EBP1 serve as hallmarks of mTOR signaling activation (43). Multiple studies (18, 26, 28) suggest that specific TCM interventions, such as Massage and Shenqi Compound may target key nodes in this pathway for activation, potentially enhancing protein synthesis in their respective models (see **Supplementary Material Table S5** for detailed mechanisms) (18, 26, 28).

The FoxO regulatory factor family plays a key role in regulating skeletal muscle protein degradation by activating the ubiquitin-proteasome system and autophagy pathways to promote muscle protein breakdown (7). Muscle Atrophy F-box Protein (Atrogin-1) and Muscle RING-Finger Protein-1 (MuRF1), as two major E3 ubiquitin ligases in the ubiquitin-proteasome system, are core regulators of muscle atrophy (44). Studies indicate that mice lacking Atrogin-1 or MuRF1 resist denervation-induced muscle atrophy (45). Intracellular signaling pathways (including FoxO, NF-κB, STAT, and Smad) can jointly activate the proteolytic pathway by regulating Atrogin-1 and MuRF1 expression, thereby accelerating protein degradation (37). Tomoaki et al. (16) proposed that the mechanism by which Juzentaihoto improves DS might be associated with the inhibition of the ubiquitin proteasome degradation pathway. Similarly, both Saikokeishikankyoto and Morinda officinalis root extract have been reported to attenuate muscle atrophy, possibly by targeting this pathway in specific animal studies (see **Supplementary Material Table S5** for specific mechanisms) (16, 33, 34).

Additionally, Zhang et al. (17) and Ma et al. (24) reported that the Zhimu-Huangbai Herb-Pair and Total Astragalus saponins may not only activate the PI3K/Akt/mTOR pathway to upregulate synthesis-related proteins but also inhibit degradation factors FoxO1 and Murf1, suggesting a bidirectional regulation of protein homeostasis by these specific agents (17, 24). Xv et al. (29) also observed that Osteoking may bidirectionally regulate muscle homeostasis, potentially by upregulating Myogenic Differentiation 1 (MyoD) and downregulating FBXO32 (see **Supplementary Table S5** for detailed mechanisms)

#### 4.2.2 Improvement of the pathological microenvironment to indirectly regulate protein synthesis and degradation

At the level of the DS pathological microenvironment, key pathological factors—including insulin resistance (IR), chronic inflammation, oxidative stress, and mitochondrial damage—appear to interact synergistically, forming a vicious cycle that collectively disrupts protein turnover balance. IR serves as a key driver in this process. As the primary target organ for insulin action, skeletal muscle that exhibits IR can induce sarcopenia through mechanisms such as activating the WW Domain Containing E3 Ubiquitin Protein Ligase 1/Kruppel-Like Factor 15 (WWP1/KLF15) protein degradation pathway and inhibiting the IGF1-Akt-mTOR protein synthesis pathway (37, 46). Chronic low-grade inflammation, a hallmark of T2DM, involves inflammatory mediators such as TNF-α, IL-6, and IL-1. These are thought to activate the ubiquitin-proteasome system through pathways like NF-κB while inhibiting the PI3K-Akt-mTOR synthetic pathway. This results in the downregulation of anabolic processes and upregulation of catabolic processes, leading to loss of muscle mass and impaired function (47–49). Oxidative stress is activated early in muscle atrophy, potentially activating the protein degradation system by increasing reactive oxygen species (ROS) and inhibiting the Akt/mTOR pathway and its downstream targets. This affects protein synthesis and degradation rates, impairing muscle regeneration (50–52). Furthermore, mitochondrial dysfunction—a core mechanism of DS—leads to insufficient ATP production, ROS surges, and kinetic abnormalities, which form the basis for insulin resistance and muscle atrophy, ultimately triggering metabolic disorders and contractile dysfunction (53, 54). These combined mechanisms result in protein turnover imbalance in T2DM patients, manifesting as progressive decline in muscle mass and function. Research suggests that certain TCM interventions may improve these pathways through multi-targeted actions. For instance, formulations such as Juzentaihoto, Dahuang Tangluo Pill, and Jianpi Fang, along with massage therapy, may mitigate IR through distinct mechanisms relevant to their composition and the models tested (16, 20, 21, 32). Others, like Buyang Huanwu tang, Osteoking, and mulberry leaf extract, exhibit anti-inflammatory and antioxidant effects, which may contribute to reducing chronic inflammation and oxidative stress-induced muscle damage (22, 29, 31). Additionally, herbs and formulas such as Astragulus embranaceus (Fisch.) Bge-Dioscorea opposita Thunb herb pair and Qinlian Jiangxia Decoction have been observed to enhance mitochondrial function (15, 25). Together, these findings suggest that these specific agents may help optimize the muscle metabolic microenvironment (see **Supplementary Material Table S5** for detailed mechanisms).

#### 4.2.3 Promotion of muscle structure repair and functional remodeling

At the level of muscle structure and function, specific TCM modalities appear to exert direct effects in promoting regeneration and repair. Skeletal muscle fibers are primarily classified into slow-twitch oxidative (Type I) and fast-twitch oxidative (Type II) types, with their functional differences largely determined by myosin isoforms (55, 56). Type I fibers contract slowly with low force but exhibit strong fatigue resistance, facilitating structural maintenance; Type II fibers contract rapidly with high force but are prone to fatigue (57). Massage has been reported to facilitate the beneficial conversion of Type II fibers to Type I fibers, thereby improving skeletal muscle pathology (18, 19). Shenqi Compound and massage may also activate satellite cells and inhibits fibrosis, potentially enhancing muscle microstructure (19, 27). Mulberry leaf extract and Osteoking appear to play unique roles in regulating glucose and lipid metabolism and suppressing ferroptosis, which may further protect skeletal muscle cell integrity (see **Supplementary Material Table S5** for detailed mechanisms) (29, 31).

**Figure 8:**
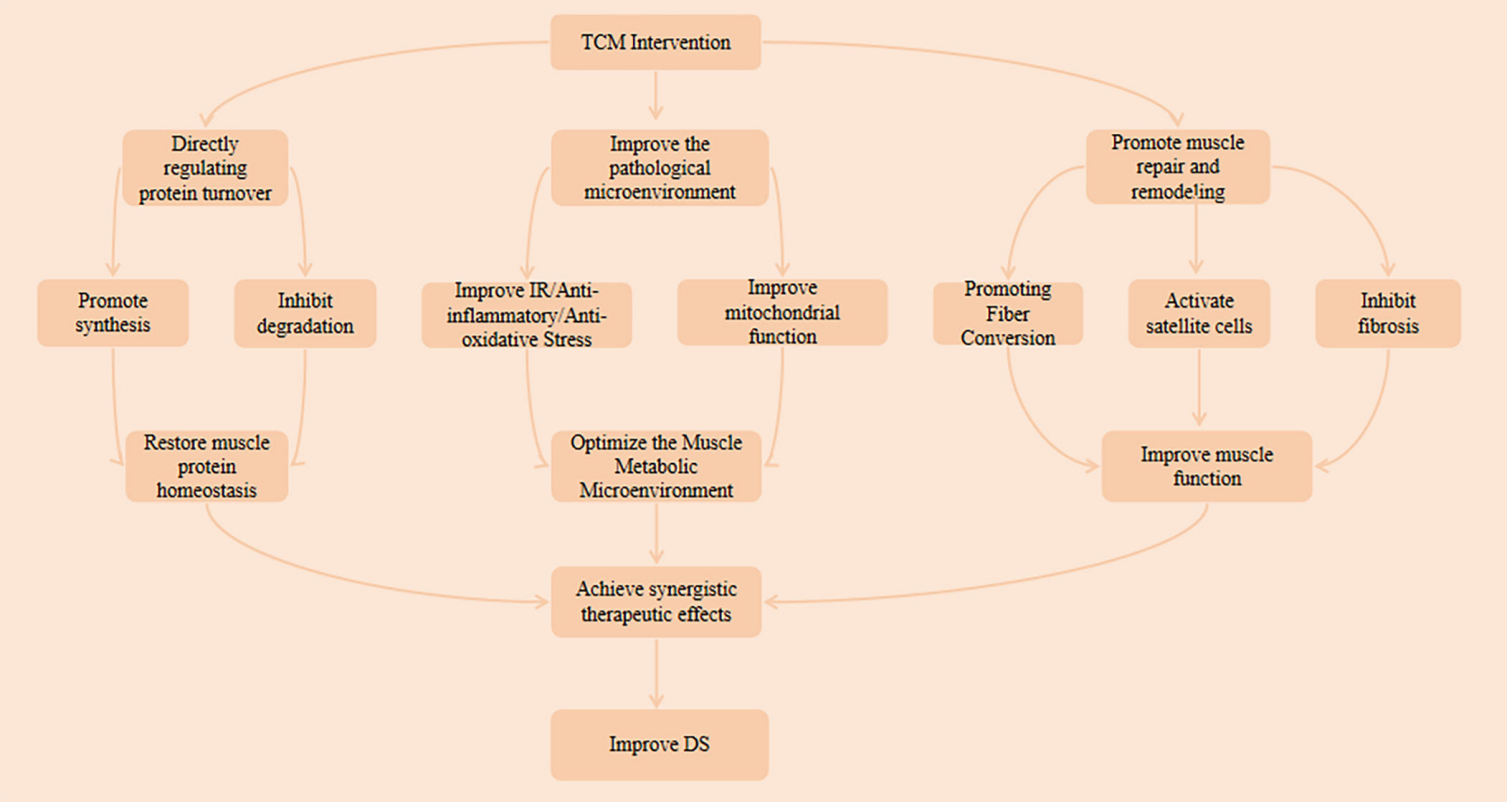
Mechanisms of traditional Chinese medicine for diabetic sarcopenia.

In summary, the reviewed TCM interventions for DS appear to act not only on core pathways such as those governing protein metabolic balance and insulin resistance but also likely modulate skeletal muscle homeostasis (promoting myofibrillar type conversion, activating satellite cells, and inhibiting fibrosis), demonstrating multi-pathway and holistic therapeutic characteristics. The proposed comprehensive mechanism of action of these TCM treatments for DS is illustrated in **Figure 8**.

### 4.3 Analysis of heterogeneity sources and assessment of evidence robustness

High heterogeneity was observed across multiple outcomes in this study, primarily stemming from differences in models and animal strains. Analysis of body weight revealed that different pathological states determined the direction of the effect, suggesting that TCM may possess a bidirectional regulatory characteristic dependent on the organism’s metabolic status. Heterogeneity in gastrocnemius muscle weight, muscle cross-sectional area, and blood glucose was largely concentrated within specific strains (e.g., C57BL/6J), indicating significant variability in response to intervention or substantial background genetic interference in this strain. Heterogeneity in grip strength was not fully explained by animal species, which may be attributed to insufficient standardization of measurement methods and a high proportion of small-sample studies.

Although limited by the number of included studies, meta-regression suggested that factors such as pathological state, intervention type, and treatment duration have potential impacts on effect sizes. However, high residual heterogeneity persisted across all models, implying that experimental details insufficiently reported in the existing literature (such as housing conditions) may be significant hidden sources of interference.

Regarding robustness, most primary outcomes showed consistent effect directions in sensitivity analyses. Furthermore, the restriction to high-quality studies further supported the reliability of the results for gastrocnemius muscle weight and blood glucose. Notably, heterogeneity for body weight decreased significantly after excluding low-quality studies, identifying study quality as a key confounding factor. It should be noted that the results for grip strength demonstrated insufficient stability, warranting cautious interpretation.

Regarding publication bias, although some outcomes suggested the presence of small-study effects, effect sizes remained substantially unchanged after adjustment using the trim-and-fill method, indicating good overall credibility of the evidence. While the effect size for gastrocnemius muscle weight decreased after adjustment, it remained statistically significant, suggesting that although the original results may have been somewhat overestimated, the conclusion remains valid.

Overall, despite heterogeneity caused by differences in models and methodologies, multiple robustness tests support the reliability of the study’s main conclusions. Future research should focus on standardizing model construction protocols and improving the reporting of experimental details to generate more reliable and translatable preclinical evidence.

### 4.4 Limitations

1) Limited sample size and methodological quality: The sample sizes of the included studies were generally small, and reporting on randomization, allocation concealment, and blinding (for both caregivers and outcome assessors) was universally unclear across all studies. These methodological flaws not only compromise the reliability of the evidence but may also exert a directional influence on the results: the lack of allocation concealment predisposes the studies to selection bias, while the absence of blinding may lead to performance bias and detection bias. In animal research, such biases have been proven to systematically overestimate the effect sizes of interventions. Consequently, the pooled effect estimates presented in this meta-analysis may be higher than the true therapeutic effects, necessitating a cautious interpretation of the conclusions; 2) Potential language bias: The included studies were primarily sourced from Chinese databases, with a low proportion of English literature, which may compromise the comprehensiveness of the results; 3) Variations in TCM formulas and interventions: Significant differences exist across studies regarding herbal ingredients, dosages, and preparation methods. These variations increase inter-study incomparability and heterogeneity; 4) Inconsistent disease severity: Differences in the duration and severity of the disease in animal models may affect the stability of the effect sizes; 5) High heterogeneity of outcome measures: Although subgroup analyses identified pathological state and animal strain as primary confounding factors, residual heterogeneity remains high. This likely stems from insufficiently reported experimental conditions (e.g., housing environment, dosage details, and detection methods); 6) Limitations in clinical extrapolation: Due to differences in pathological complexity and metabolic characteristics between animal models and humans, caution is required when translating these results to clinical practice.

### 4.5 Future perspectives

1) Rigorous methodological implementation: Future studies must strictly adhere to the SYRCLE tool and ARRIVE guidelines, strengthening the implementation and reporting of critical methodological steps such as randomization and blinding, while reasonably expanding sample sizes. Concurrently, animal model selection should be optimized to use models that better match human metabolic characteristics and disease stages, ensuring a systematic evaluation of intervention effects and guaranteeing result reliability and stability; 2) Promoting TCM standardization and mechanistic research: It is recommended to gradually unify the ingredients, dosages, and preparation processes of TCM formulas in both preclinical and clinical studies to enhance comparability and reduce heterogeneity. Mechanistic research should transcend simple efficacy verification to systematically elucidate multi-target interaction networks. In particular, the causal mechanisms underlying model-dependent effects, such as the bidirectional regulation of body weight, should be explored in depth; 3) Clinical translation and implementation strategies: Building upon reliable preclinical evidence, rigorously designed clinical trials should be advanced. It is recommended to stratify patients based on metabolic phenotypes (e.g., obesity status) and adopt multi-center, randomized, double-blind designs. Comprehensive assessments should include multidimensional indices such as muscle mass, grip strength, and physical function to provide high-level evidence-based support for the efficacy and safety of TCM in treating diabetic sarcopenia.

## 5 Conclusion

Current preclinical evidence suggests that specific TCM interventions may show potential in alleviating Diabetic Sarcopenia in animal models by improving muscle mass, strength, and metabolic function. Certain formulations appeared to exhibit a bidirectional regulation of body weight, which may reflect a context-dependent metabolic impact. However, due to the prevalent lack of blinding and allocation concealment in the included studies, the reported efficacy may be systematically overestimated. Consequently, current evidence remains preliminary. Future research must strictly adhere to ARRIVE guidelines and standardize interventions to generate robust preclinical data, paving the way for high-quality clinical trials.

## Data Availability

The datasets presented in this study can be found in online repositories. The names of the repository/repositories and accession number(s) can be found in the article/**Supplementary Material**.
