# Supplementary material for: Therapeutic effect of traditional Chinese medicine on diabetic sarcopenia: a systematic review and meta-analysis of preclinical studies
Source: Front Endocrinol (Lausanne). 2026 Jan 12;16:1647271. doi: 10.3389/fendo.2025.1647271 (PMC12832245; doi:10.3389/fendo.2025.1647271)

# Supplementary Table S1 Literature Search Strategy

**Pubmed**

| Search number | Query | Results |
| --- | --- | --- |
| #1 | **"Diabetes Mellitus"[Mesh] OR "Diabetes Mellitus"[Title/Abstract] OR diabetic[Title/Abstract])** | 751,221 |
| #2 | **("Sarcopenia"[Mesh]) OR ("Muscular Atrophy"[Mesh]) OR (sarcopenia[Title/Abstract]) OR ("muscle loss"[Title/Abstract]) OR ("muscular atrophy"[Title/Abstract]) OR ("skeletal muscle atrophy"[Title/Abstract]) OR ("muscle wasting"[Title/Abstract])** | 55,268 |
| #3 | **("Medicine, Chinese Traditional"[Mesh]) OR ("Drugs, Chinese Herbal"[Mesh]) OR ("Acupuncture Therapy"[Mesh]) OR ("massage"[Mesh]) OR ("Traditional Chinese Medicine"[Title/Abstract]) OR (TCM[Title/Abstract]) OR ("Chinese herb*"[Title/Abstract]) OR (herb*[Title/Abstract]) OR (decoction[Title/Abstract]) OR (formula[Title/Abstract]) OR ("herbal formula"[Title/Abstract]) OR (granule[Title/Abstract]) OR (capsule[Title/Abstract]) OR (acupuncture[Title/Abstract]) OR (electroacupuncture[Title/Abstract]) OR (acupunct*[Title/Abstract]) OR (moxibustion[Title/Abstract]) OR (tuina[Title/Abstract]) OR (massage[Title/Abstract]) OR (polysaccharide[Title/Abstract]) OR (extract[Title/Abstract]) OR (polyphenol[Title/Abstract]) OR (supplement[Title/Abstract])** | 963,037 |
| #4 | #1 AND #2 AND #3 | 67 |

**Embase**

| Search number | Query | Results |
| --- | --- | --- |
| #1 | **'diabetes mellitus'/exp OR 'diabetes mellitus':ti,ab OR diabetic:ti,ab** | 1,624,396 |
| #2 | **'sarcopenia'/exp OR 'muscle atrophy'/exp OR sarcopenia:ti,ab OR 'muscle loss':ti,ab OR 'muscular atrophy':ti,ab OR 'skeletal muscle atrophy':ti,ab OR 'muscle wasting':ti,ab** | 104,687 |
| #3 | **'chinese medicine'/exp OR 'chinese drug'/exp OR 'acupuncture'/exp OR 'massage'/exp OR 'Traditional Chinese Medicine':ti,ab,kw OR TCM:ti,ab,kw OR 'Chinese herb*':ti,ab,kw OR herb*:ti,ab,kw OR decoction:ti,ab,kw OR formula:ti,ab,kw OR 'herbal formula':ti,ab,kw OR granule:ti,ab,kw OR capsule:ti,ab,kw OR acupuncture:ti,ab,kw OR electroacupuncture:ti,ab,kw OR acupunct*:ti,ab,kw OR moxibustion:ti,ab,kw OR tuina:ti,ab,kw OR massage:ti,ab,kw OR polysaccharide:ti,ab,kw OR extract:ti,ab,kw OR polyphenol:ti,ab,kw OR supplement:ti,ab,kw** | 1,376,206 |
| #4 | #1 AND #2 AND #3 | 274 |

**Web of science**

| Search number | Query | Results |
| --- | --- | --- |
| #1 | **TS=("Diabetes Mellitus" OR diabetic)** | 488,535 |
| #2 | **TS=("Sarcopenia" OR "Muscular Atrophy" OR "muscle loss" OR "muscular atrophy" OR "skeletal muscle atrophy" OR "muscle wasting")** | 52,388 |
| #3 | **TS=("Medicine, Chinese Traditional" OR "Drugs, Chinese Herbal" OR "Acupuncture Therapy" OR massage OR "Traditional Chinese Medicine" OR TCM OR "Chinese herb*" OR herb* OR decoction OR formula OR "herbal formula" OR granule OR capsule OR acupuncture OR electroacupuncture OR acupunct* OR moxibustion OR tuina OR polysaccharide OR extract OR polyphenol OR supplement)** | 2,462,835 |
| #4 | #1 AND #2 AND #3 | 115 |

**Cochrane**

| Search number | Query | Results |
| --- | --- | --- |
| #1 | **MeSH descriptor: [Diabetes Mellitus] explode all trees** | 47,647 |
| #2 | **"diabetes mellitus":ti,ab OR diabetic:ti,ab** | 68,033 |
| #3 | **#1 OR #2** | 90,403 |
| #4 | MeSH descriptor: [Sarcopenia] explode all trees | 1,089 |
| #5 | MeSH descriptor: [Muscular Atrophy] explode all trees | 1,612 |
| #6 | sarcopenia:ti,ab OR "muscle loss":ti,ab OR "muscular atrophy":ti,ab OR "skeletal muscle atrophy":ti,ab OR "muscle wasting":ti,ab | 4,112 |
| #7 | #4 OR #5 OR #6 | 4,788 |
| #8 | MeSH descriptor: [Medicine, Chinese Traditional] explode all trees | 1,868 |
| #9 | MeSH descriptor: [Drugs, Chinese Herbal] explode all trees | 4,776 |
| #10 | MeSH descriptor: [Acupuncture Therapy] explode all trees | 7,335 |
| #11 | MeSH descriptor: [Massage] explode all trees | 1,777 |
| #12 | "Traditional Chinese Medicine":ti,ab OR TCM:ti,ab OR Chinese herb*:ti,ab OR herb*:ti,ab OR decoction:ti,ab OR formula:ti,ab OR "herbal formula":ti,ab OR granule:ti,ab OR capsule:ti,ab OR acupuncture:ti,ab OR electroacupuncture:ti,ab OR acupunct*:ti,ab OR moxibustion:ti,ab OR tuina:ti,ab OR massage:ti,ab OR polysaccharide:ti,ab OR extract:ti,ab OR polyphenol:ti,ab OR supplement:ti,ab | 136,486 |
| #13 | #8 OR #9 OR #10 #11 OR #12 | 139,525 |
| #14 | #3 AND #7 AND #13 | 11 |

**CNKI**

| Search number | Query | Results |
| --- | --- | --- |
| #1 | **(SU='糖尿病') AND (SU='肌少症' OR SU='肌肉减少症' OR SU='肌萎缩' OR SU='肌肉萎缩' OR SU='骨骼肌萎缩') AND (SU='中医药' OR SU='中医' OR SU='中药' OR SU='中草药' OR SU='草药' OR SU='针灸' OR SU='针刺' OR SU='电针' OR SU='艾灸' OR SU='推拿' OR SU='按摩' OR SU='汤剂' OR SU='方剂' OR SU='复方' OR SU='颗粒' OR SU='胶囊' OR SU='多糖' OR SU='提取物' OR SU='多酚' OR SU='补充剂')** | 83 |

**VIP**

| Search number | Query | Results |
| --- | --- | --- |
| #1 | **U=(糖尿病) AND U=(肌少症 OR 肌肉减少症 OR 肌萎缩 OR 肌肉萎缩 OR 骨骼肌萎缩) AND U=(中医药 OR 中医 OR 中药 OR 中草药 OR 草药 OR 针灸 OR 针刺 OR 电针 OR 艾灸 OR 推拿 OR 按摩 OR 汤剂 OR 方剂 OR 复方 OR 颗粒 OR 胶囊 OR 多糖 OR 提取物 OR 多酚 OR 补充剂)** | 360 |

**WanFang**

| Search number | Query | Results |
| --- | --- | --- |
| #1 | **(主题:("糖尿病") AND 主题:("肌少症" OR "肌肉减少症" OR "肌萎缩" OR "肌肉萎缩" OR "骨骼肌萎缩") AND 主题:("中医药" OR "中医" OR "中药" OR "中草药" OR "草药" OR "针灸" OR "针刺" OR "电针" OR "艾灸" OR "推拿" OR "按摩" OR "汤剂" OR "方剂" OR "复方" OR "颗粒" OR "胶囊" OR "多糖" OR "提取物" OR "多酚" OR "补充剂")** | 301 |

| **Supplementary Table S2** Univariate meta-regression analysis of potential sources of heterogeneity for all outcomes | | | | | | | | |
| --- | --- | --- | --- | --- | --- | --- | --- | --- |
| Outcome indicator | Covariate | No. of Studies | Coefficient | Std. Err. | t value | P value | 95% CI | Adj R² (%) |
| Body Weight Outcome | Pathological State | 14 | -2.186 | 1.127 | -1.94 | 0.076 | -4.641, 0.269 | 25.41 |
|  | Animal Species | 14 | -0.44 | 0.269 | -1.64 | 0.128 | -1.026, 0.146 | 10.38 |
|  | Intervention Category | 14 | 0.18 | 0.609 | 0.29 | 0.773 | -1.148, 1.507 | -13.77 |
|  | Disease Model | 14 | -0.45 | 0.645 | -0.7 | 0.499 | -1.856, 0.957 | -4.99 |
|  | Treatment Duration | 14 | -0.052 | 0.609 | -0.08 | 0.934 | -1.380, 1.276 | -13.38 |
| Gastrocnemius muscle weight | Pathological State | 11 | -0.254 | 1.498 | -0.17 | 0.869 | -3.642, 3.134 | -17.28 |
|  | Animal Species | 11 | -0.346 | 0.368 | -0.94 | 0.372 | -1.178, 0.486 | -2.29 |
|  | Intervention Category | 11 | -0.905 | 0.499 | -1.81 | 0.103 | -2.035, 0.225 | 25.44 |
|  | Disease Model | 11 | 0.843 | 0.603 | 1.4 | 0.196 | -0.521, 2.207 | 13.35 |
|  | Treatment Duration | 11 | -0.05 | 0.603 | -0.08 | 0.935 | -1.414, 1.313 | -17.77 |
| Grip strength | Animal Species | 5 | -1.314 | 0.665 | -1.98 | 0.142 | -3.429, 0.801 | 52.67 |
|  | Intervention Category | 5 | -0.862 | 0.608 | -1.42 | 0.251 | -2.798, 1.073 | 18.81 |
|  | Treatment Duration | 5 | -0.303 | 0.868 | -0.35 | 0.75 | -3.064, 2.458 | -44.35 |
| Muscle cross-sectional area | Pathological State | 8 | 0.062 | 1.63 | 0.04 | 0.971 | -3.926, 4.049 | -35.93 |
|  | Animal Species | 8 | -0.972 | 0.815 | -1.19 | 0.278 | -2.966, 1.023 | -8.3 |
|  | Intervention Category | 8 | -0.389 | 1.383 | -0.28 | 0.788 | -3.773, 2.995 | -38.46 |
|  | Disease Model | 8 | -0.561 | 0.859 | -0.65 | 0.538 | -2.662, 1.541 | -35.05 |
|  | Treatment Duration | 8 | -1.053 | 0.654 | -1.61 | 0.158 | -2.652, 0.547 | 40.14 |
| Blood glucose | Pathological State | 16 | -0.705 | 1.771 | -0.4 | 0.697 | -4.502, 3.093 | -11.95 |
|  | Animal Species | 16 | -0.404 | 0.386 | -1.05 | 0.313 | -1.232, 0.424 | -6.51 |
|  | Intervention Category | 16 | 0.341 | 0.695 | 0.49 | 0.631 | -1.150, 1.832 | -9.66 |
|  | Disease Model | 16 | 0.084 | 0.822 | 0.1 | 0.92 | -1.678, 1.846 | -11.83 |
|  | Treatment Duration | 16 | -0.74 | 0.427 | -1.73 | 0.105 | -1.657, 0.176 | 24.66 |
| **Annotations:** Std. Err., Standard Error; CI, Confidence Interval; Adj R², Adjusted R-squared. The coefficient represents the estimated change in the effect size (e.g., SMD or MD) per unit change in the covariate. A negative Adjusted R² value indicates that the meta-regression model explains less heterogeneity than would be expected by chance alone, and the interpretation of the variance explained should be treated with caution. | | | | | | | | |

| **Supplementary Table S3** Results of the sensitivity analysis using the leave-one-out method | | |
| --- | --- | --- |
| Outcome Indicator | Excluded studies | Pooled effect size (95% CI) |
| Body weight | None (Combined) | 0.33 (-0.40 to 1.06) |
|  | Huang (2011) | 0.08 (-0.56 to 0.73) |
|  | Meng (2023) | 0.31 (-0.47 to 1.08) |
|  | Qi (2017) | 0.27 (-0.51 to 1.06) |
|  | Shi (2023) | 0.41 (-0.37 to 1.18) |
|  | Zhao (2024b) | 0.27 (-0.50 to 1.04) |
|  | Zhong (2018) | 0.38 (-0.41 to 1.17) |
|  | Zuo (2022) | 0.38 (-0.42 to 1.17) |
|  | Tomoaki (2022) | 0.22 (-0.54 to 0.97) |
|  | Wang (2024) | 0.20 (-0.54 to 0.95) |
|  | Xv (2022) | 0.34 (-0.48 to 1.16) |
|  | Zhang (2014) | 0.29 (-0.51 to 1.10) |
|  | Zhao (2024a) | 0.53 (-0.16 to 1.22) |
|  | Ou (2022) | 0.51 (-0.20 to 1.21) |
|  | Wang (2021) | 0.47 (-0.27 to 1.20) |
| Gastrocnemius muscle weight | None (Combined) | 2.00 (1.18 to 2.83) |
|  | Guo (2024) | 1.80 (1.00 to 2.61) |
|  | Meng (2023) | 1.83 (1.01 to 2.65) |
|  | Ma (2024) | 2.14 (1.21 to 3.06) |
|  | Zhao (2024b) | 2.12 (1.21 to 3.03) |
|  | Zuo (2022) | 2.20 (1.28 to 3.12) |
|  | Zhong (2018) | 1.64 (0.94 to 2.34) |
|  | Tomoaki (2022) | 2.08 (1.17 to 2.98) |
|  | She (2023) | 1.89 (1.04 to 2.73) |
|  | Zhang (2014) | 2.22 (1.29 to 3.15) |
|  | Xv (2022) | 2.22 (1.29 to 3.15) |
|  | Ou (2022) | 1.99 (1.11 to 2.87) |
| Grip strength | None (Combined) | 1.70 (0.55 to 2.85) |
|  | Xv (2022) | 2.17 (0.82 to 3.52) |
|  | Zhang (2014) | 1.87 (0.29 to 3.45) |
|  | Meng (2023) | 1.52 (0.26 to 2.77) |
|  | Guo (2024) | 1.10 (0.25 to 1.94) |
|  | Zuo (2022) | 2.06 (0.51 to 3.61) |
| Muscle cross-sectional area | None (Combined) | 2.93 (1.80 to 4.06) |
|  | She (2023) | 2.78 (1.58 to 3.98) |
|  | Tomoaki (2022) | 3.10 (1.78 to 4.42) |
|  | Zhang (2014) | 2.85 (1.60 to 4.09) |
|  | Zhao (2024b) | 3.19 (1.87 to 4.51) |
|  | Zhong (2025) | 2.58 (1.53 to 3.63) |
|  | Xv (2022) | 3.24 (2.13 to 4.34) |
|  | Wang (2021) | 2.63 (1.55 to 3.71) |
|  | Ou (2022) | 3.18 (1.84 to 4.51) |
| Blood glucose | None (Combined) | -3.38 (-4.37 to -2.40) |
|  | Tomoaki (2022) | -3.46 (-4.50 to -2.41) |
|  | Zhang (2014) | -3.62 (-4.69 to -2.54) |
|  | Zhao (2024a) | -3.14 (-4.08 to -2.20) |
|  | Sun (2025) | -3.54 (-4.59 to -2.48) |
|  | Wang (2024) | -3.00 (-3.90 to -2.10) |
|  | Zhu (2015) | -3.29 (-4.29 to -2.28) |
|  | Ma (2024) | -3.14 (-4.10 to -2.19) |
|  | Zhao (2024b) | -3.36 (-4.38 to -2.34) |
|  | Zhong (2018) | -3.59 (-4.66 to -2.52) |
|  | Zuo (2022) | -3.62 (-4.61 to -2.63) |
|  | Meng (2023) | -3.29 (-4.29 to -2.28) |
|  | Huang (2011) | -3.54 (-4.61 to -2.48) |
|  | Shi (2023) | -3.35 (-4.37 to -2.33) |
|  | Qi (2017) | -3.42 (-4.46 to -2.37) |
|  | Wang (2021) | -3.38 (-4.40 to -2.35) |
|  | Ou (2022) | -3.50 (-4.56 to -2.45) |
| **Annotations:** Pooled effect sizes are presented as standardized mean difference (SMD) with 95% confidence intervals (CI). A positive SMD indicates an effect favoring the intervention for body weight, muscle weight, and cross-sectional area; a negative SMD indicates a reduction in blood glucose. "None (Combined)" represents the overall meta-analysis result for each outcome. | | |
|  |  |  |
|  |  |  |

| **Supplementary Table S4** Assessment of publication bias using Egger’s test and the Trim-and-Fill method for all outcomes | | | | | | |
| --- | --- | --- | --- | --- | --- | --- |
| Outcome | Number of Studies | Egger's Test  P-value | Egger's Bias Coef. (95% CI) | Trim & Fill Imputed Studies | Pre-adjustment SMD (95% CI) | Post-adjustment SMD (95% CI) |
|  |  |  |  |  |  |  |
|  |  |  |  |  |  |  |
| Body Weight | 14 | 0.979 | 0.063 (-5.136, 5.262) | NA | NA | NA |
| Gastrocnemius muscle weight | 11 | < 0.001 | 5.937 (4.575, 7.299) | 2 | 2.004 (1.178, 2.830) | 1.526 (0.651, 2.401) |
| Grip Strength | 5 | 0.033 | 5.675 (0.857, 10.493) | 0 | 1.702 (0.552, 2.851) | 1.702 (0.552, 2.851) |
| Muscle cross-sectional area | 8 | 0.005 | 4.241 (1.819, 6.663) | 0 | 2.932 (1.799, 4.065) | 2.932 (1.799, 4.065) |
| Blood Glucose | 16 | < 0.001 | -5.761 (-7.257, -4.265) | 0 | -3.384 (-4.368, -2.401) | -3.384 (-4.368, -2.401) |
| **Annotations:** CI, confidence interval; SMD, standardized mean difference; CSA, cross-sectional area; NA, not applicable. Egger's test P < 0.05 indicates potential publication bias. The Trim-and-Fill method was not performed for the "Body Weight" outcome as no significant publication bias was detected (Egger's P > 0.05). For other outcomes, "0" imputed studies indicate that no missing studies were identified, and the results are considered robust. | | | | | | |
|  |  |  |  |  |  |  |
|  |  |  |  |  |  |  |

| **Supplementary Table S5** Summary of the Mechanisms of Action of TCM Interventions for Diabetic Sarcopenia from the Included Studies | | | |
| --- | --- | --- | --- |
| Study | Intervention measures | Mechanism of Action | Impact Indicators(Compared to the model group) |
| She 2023^[15]^ | Astragulus embranaceus (Fisch.) Bge-Dioscorea opposita Thunb herb pair | Activate the mTOR/PGC-1α signaling pathway to improve mitochondrial dysfunction. | Mitochondrial biogenesis markers: Elevated expression of PGC-1α (peroxisome proliferator-activated receptor gamma coactivator 1α), Nrf1 (nuclear factor erythroid 2-related factor 1), and TFAM (mitochondrial transcription factor A); Mitochondrial dynamics markers: Elevated expression of MFF (mitochondrial fission factor) and decreased expression of Mitofusin-2, a mitochondrial fusion-associated protein; AMPK (adenosine monophosphate-activated protein kinase) expression increased; PGC-1α expression increased. |
| Tomoaki 2022^[16]^ | Juzentaihoto | Improve insulin resistance; suppress inflammation; inhibit the ubiquitin-proteasome degradation pathway. | Pro-inflammatory cytokines: Tumor Necrosis Factor-α (TNF-α) and Interleukin-6 (IL-6) levels decreased; Homeostatic Model Assessment of Insulin Resistance (HOMA-IR) improved; Proteolysis-associated factors: mRNA expression levels of Atrogin-1 (MAFBX) and MuRF1 (Muscle Unnamed Finger Protein-1) decreased; Increased mRNA levels of Sirtuin1 (SIRT1). |
| Zhang 2014^[17]^ | Zhimu-Huangbai Herb-Pair | Activate the mTOR signaling pathway to promote protein synthesis and inactivate Foxo3 protein to inhibit protein degradation. | Elevated serum insulin-like growth factor 1 (IGF-1);Elevated levels of phosphorylated proteins: p-Akt (protein kinase B), p-mTOR (mammalian target of rapamycin), p-Raptor (key component of mTOR complex 1), p-S6K1 (ribosomal protein S6 kinase 1); reduced expression of Foxo3. |
| Guo 2024^[18]^ | Massage | Upregulation of the IGF-1/PI3K/Akt signaling pathway promotes protein synthesis；Promote the conversion of type II (fast-twitch) muscle fibers to type I (slow-twitch) muscle fibers. | IGF-1, p-PI3K, p-Akt, p-mTOR, and p70s6k protein and mRNA expression were significantly upregulated; ATPase staining revealed an increased proportion of type I muscle fibers. |
| Meng 2023^[19]^ | Massage | Promotes the conversion of skeletal muscle type II fibers to type I fibers, enhances the expression of myogenic determinants in skeletal muscle, and suppresses myostatin expression, thereby promoting the myogenic differentiation of muscle satellite cells. | An increase in the number of Type I muscle fibers (slow-twitch fibers) and a decrease in the number of Type II muscle fibers (fast-twitch fibers); Increase the protein and mRNA expression levels of MyoD (Myo-proliferation-inducing protein). Decrease the protein and mRNA expression levels of MSTN (Myostatin, also known as GDF-8). |
| Zhao 2024a^[20]^ | Dahuang Tangluo Pill | Regulate the RXRA/TNF-α/GLUT4 pathway to improve insulin resistance. | The insulin resistance index decreased while the insulin sensitivity index increased; Glut4, Rxra mRNA, and protein expression were significantly upregulated; Tnfa mRNA and protein expression were significantly downregulated. |
| Sun 2025^[21]^ | Massage | Reduce collagen deposition in the extracellular matrix of skeletal muscle cells; reduce insulin resistance. | Reduced expression of type I and type III collagen mRNA and protein; decreased fasting blood glucose and serum insulin levels. |
| Wang 2024^[22]^ | Buyang Huanwu tang | Regulating Mitochondrial Dynamics;Anti-inflammatory Effects;Counteracting Oxidative Stress. | Mitochondrial dynamics-related proteins (Mfn2 elevated, Drp1 decreased); Inflammatory mediators (IL-6, TNF-α decreased); Oxidative stress markers (ROS decreased). |
| Zhu 2015^[23]^ | Shenqi Compound | Regulate sugar and lipid metabolism, protect blood vessels. | Blood glucose, total cholesterol, triglycerides, low-density lipoprotein cholesterol, and high-density lipoprotein cholesterol levels (all abnormally elevated) decreased; aortic intimal edema was reduced, and endothelial cell detachment decreased. |
| Ma 2024^[24]^ | Total Astragalus saponins | Activate the PI3K/Akt/mTOR pathway to promote skeletal muscle protein synthesis; inhibit the PI3K/Akt/FoxO1 pathway to reduce muscle protein degradation (decrease Murf1 expression). | Protein expression: Elevated levels of PI3K, p-Akt, mTOR, and S6K1; decreased levels of FoxO1 and Murf1. mRNA expression: Elevated levels of PI3K, Akt, and mTOR. |
| Zhao 2024b^[25]^ | Qinlian Jiangxia Decoction | Regulate AMPK-mediated mitochondrial biogenesis and autophagy to improve mitochondrial function; alleviate oxidative stress. | AMPKα, p-AMPKα, PGC-1α, NRF-1, and TFAM expression were upregulated; PINK1 and Parkin expression, as well as their colocalization with TOM20, were enhanced; MDA content decreased and SOD activity increased. |
| Zhong 2018^[26]^ | Shenqi compound | Activate the IGF-1/PI3K/Akt/p70s6k signaling pathway | Elevated serum IGF-1 levels; increased protein expression levels (elevated PI3K expression, elevated Akt expression, elevated p70s6k expression) |
| Zhong 2025^[27]^ | Shenqi compound | Inhibit the Wnt/β-catenin signaling pathway; regulate the TGF-β1/Smad pathway; restore muscle satellite cell activity. | Downregulation of fibrosis-related genes (β-catenin, Axin2, Smad3); Upregulation of Pax7, a marker of myoblasts. |
| Zuo 2022^[28]^ | Campanumoea javanica Bl | Activate the PI3K/AKT/mTOR signaling pathway and regulate the synthesis of corresponding proteins. | Promotes mRNA Upregulation: mRNA expression of mTOR (mammalian target of rapamycin), AKT (protein kinase B), PI3K (phosphatidylinositol 3-kinase), S6K (ribosomal protein S6 kinase), and 4E-BP1 (eukaryotic initiation factor 4 elongation protein-binding protein 1) is elevated. |
| Xv 2022^[29]^ | Osteoking | Inhibit oxidative stress; inhibit ferroptosis; enhance muscle synthesis and inhibit atrophy. | Levels of reactive oxygen species (ROS) and malondialdehyde (MDA) decreased; expression of ferroptosis-related proteins ELAVL1, ferritin heavy chain (FTH-1), Beclin-1, NCOA4, and LC3 decreased; expression of the myogenic differentiation antigen MyoD increased, while expression of the muscle atrophy marker FBXO32 (Atrogin-1) decreased. |
| Huang 2011^[30]^ | Mulberry leaf polysaccharide | Improve oxidative stress; reduce skeletal muscle damage. | Markers of oxidative stress: Decreased MDA levels and increased superoxide SOD levels; Markers of skeletal muscle injury: Decreased creatine kinase (CK) levels and decreased lactate dehydrogenase (LDH) levels. |
| Shi 2023^[31]^ | Mulberry leaf extract | Improve lipid metabolism; inhibit ferroptosis. | Protein and mRNA expression of ApoA1 and ApoA4 were significantly downregulated; skeletal muscle ferroptosis regulatory proteins Cp and Fth showed markedly reduced expression. |
| Qi 2017^[32]^ | Jianpi Fang | Improve skeletal muscle insulin resistance. | Elevated CLUT4 expression levels. |
| Wang 2021^[33]^ | Root extract of Morinda officinalis | Enhance mitochondrial biogenesis and myogenesis, thereby inhibiting the ubiquitin-proteasome pathway | Mitochondrial biogenesis and myogenesis enhancement: PGC-1α, SIRT1, NRF1, TFAM, MyoD, myogenin, and MHC protein expression upregulated; Muscle atrophy inhibition: Atrogin-1 protein expression downregulated. |
| Ou 2022^[34]^ | Saikokeishikankyoto | Activate the Sirt1 signalling pathway to suppress inflammation and protein degradation; Downregulate the ubiquitin ligase Atrogin-1/MuRF1 to reverse muscle atrophy. | Sirt1 signaling activation: Sirt1 expression increased; serum TNF-α decreased; Muscle protein degradation inhibition: Atrogin-1 and MuRF1 mRNA downregulated. |
| Annotations: Abbreviations used in the table: mTOR (mammalian target of rapamycin), PGC-1α (peroxisome proliferator-activated receptor gamma coactivator 1-alpha), NRF1 (nuclear respiratory factor 1), TFAM (mitochondrial transcription factor A), AMPK (AMP-activated protein kinase), TNF-α (tumor necrosis factor-alpha), IL-6 (interleukin-6), HOMA-IR (homeostatic model assessment of insulin resistance), SIRT1 (sirtuin 1), IGF-1 (insulin-like growth factor 1), Akt (protein kinase B), S6K1 (ribosomal protein S6 kinase 1), FoxO (forkhead box O), PI3K (phosphatidylinositol 3-kinase), GLUT4 (glucose transporter type 4), ROS (reactive oxygen species), MDA (malondialdehyde), SOD (superoxide dismutase), MyoD (myogenic differentiation antigen), myogenin, MHC (myosin heavy chain), MuRF1 (muscle RING-finger protein-1), MSTN (myostatin). All impact indicators represent changes compared to the model control group. | | | |


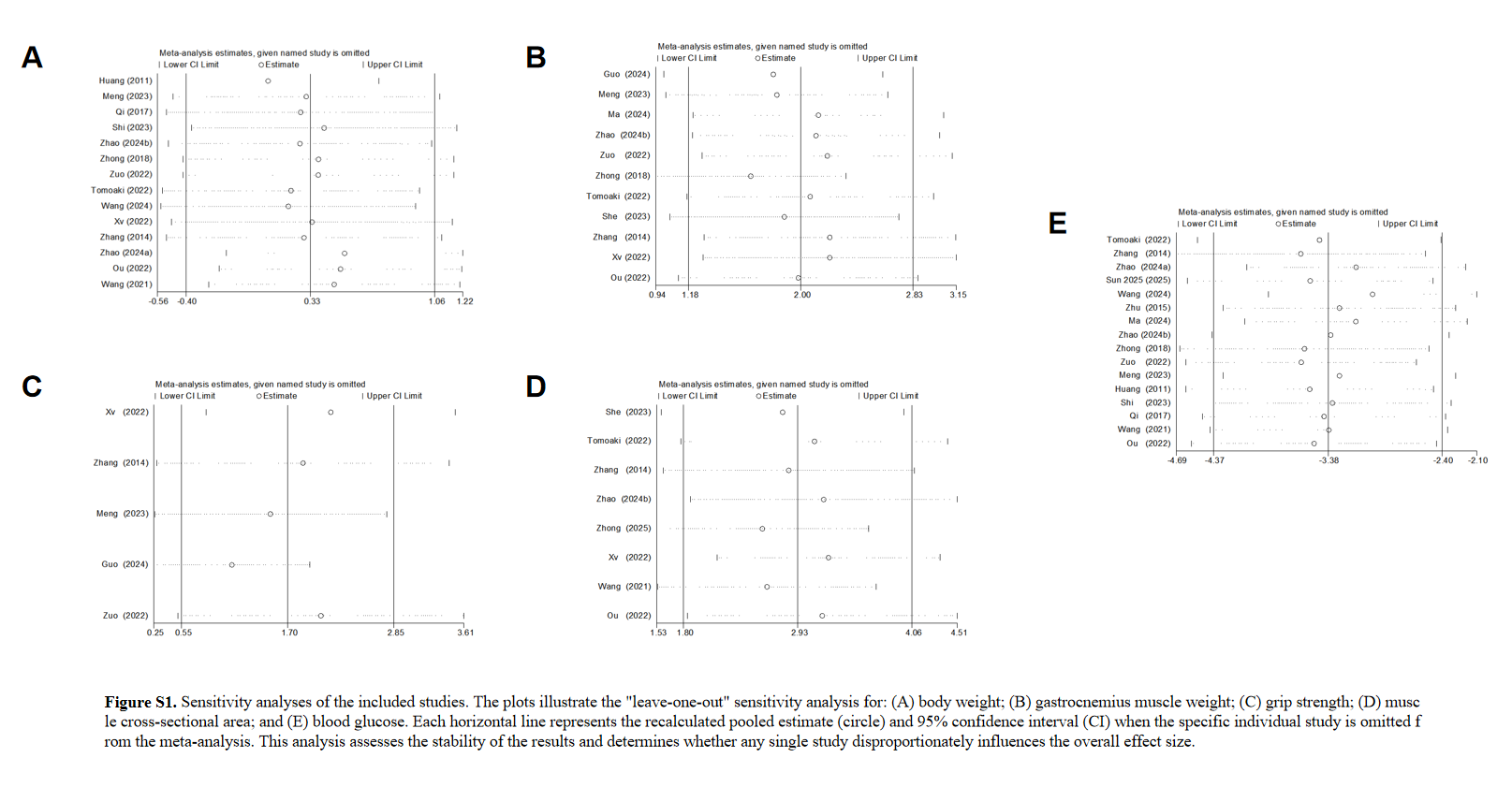

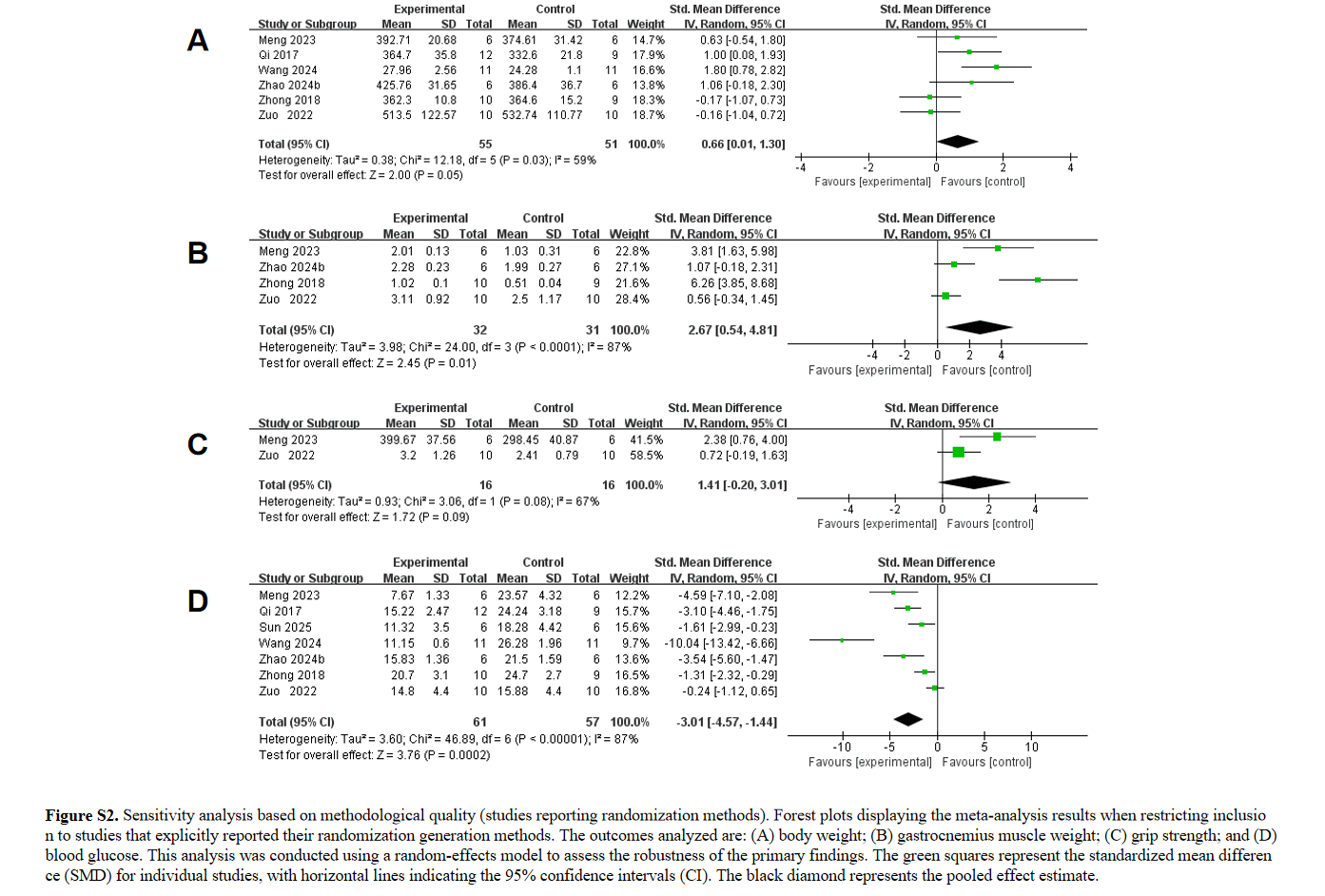


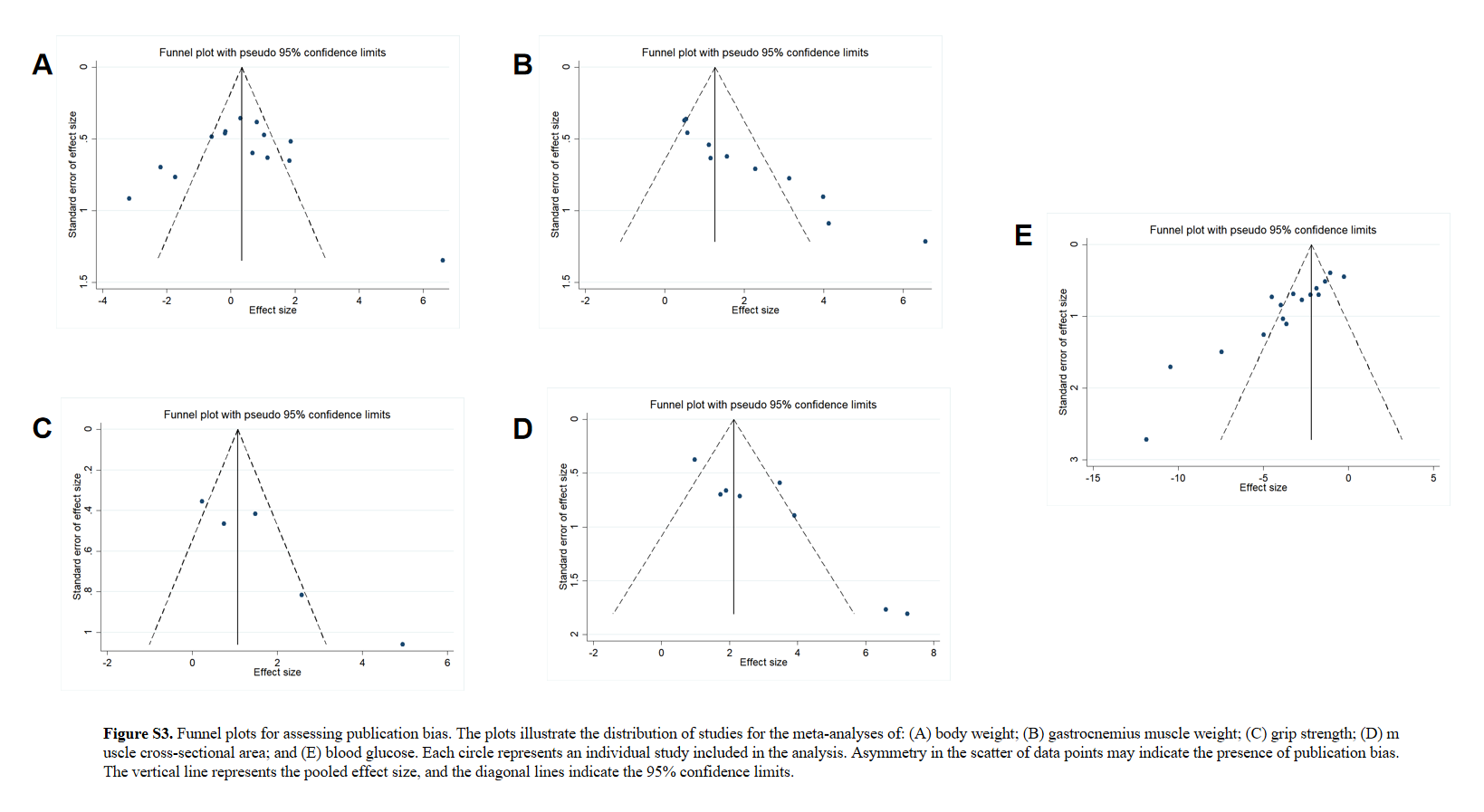

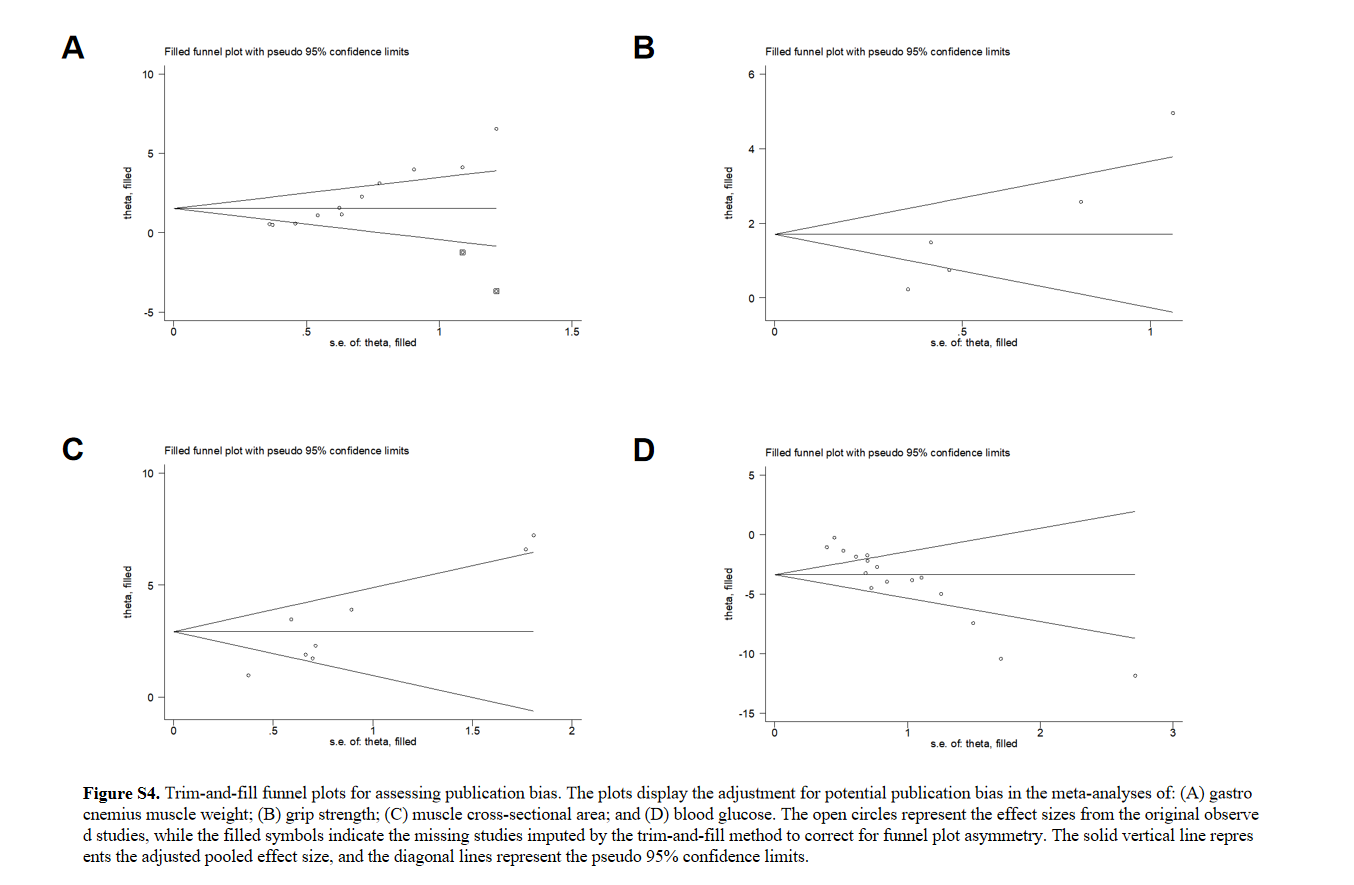

Supplement: Supplementary file 1 [file DataSheet1.docx]
