# Supplementary material for: Therapeutic effect of traditional Chinese medicine on diabetic sarcopenia: a systematic review and meta-analysis of preclinical studies
Source: Front Endocrinol (Lausanne). 2026 Jan 12;16:1647271. doi: 10.3389/fendo.2025.1647271 (PMC12832245; doi:10.3389/fendo.2025.1647271)
Supplement: Supplementary file 2 [file DataSheet2.docx]

**Identification of studies via databases, registers and other sources**

Full-text articles excluded (n=43):

Insufficient data for outcome indicators(n=5)

In vitro experiments(n=1)

Non-type 2 diabetes animal model(n=17)

Intervention method is not compliant(n=17)

Duplicate publication(n=3)

Studies included in review(n=20)

**Includeeded**

Records excluded after screening by title and abstract(n = 873)

Full-text article assessed for eligibility(n=63)

**Screening**

Records identified from databases(n=1211):

CNKI (n=83) VIP (n=360)

WanFang(n=301) Embase(n=274) Pubmed (n=67) Cochrane (n=11)

Web Of Science (n=115)

Records identified from other methods(n=5)

Duplicated records(n=280)

Records screened by title and abstract(n=936)

**Identification**

*Consider, if feasible to do so, reporting the number of records identified from each database or register searched (rather than the total number across all databases/registers).

**If automation tools were used, indicate how many records were excluded by a human and how many were excluded by automation tools.

Source: Page MJ, et al. BMJ 2021;372:n71. doi: 10.1136/bmj.n71.

This work is licensed under CC BY 4.0. To view a copy of this license, visit https://creativecommons.org/licenses/by/4.0/
